# A novel and efficient CD22 CAR-T therapy induced a robust antitumor effect in relapsed/refractory leukemia patients when combined with CD19 CAR-T treatment as a sequential therapy

**DOI:** 10.1186/s40164-022-00270-5

**Published:** 2022-03-22

**Authors:** Yu Zhang, Saisai Li, Ying Wang, Yang Lu, Yingxi Xu, Qing Rao, Huijun Wang, Haiyan Xing, Zheng Tian, Kejing Tang, Lulu Lv, Min Wang, Jianxiang Wang

**Affiliations:** 1 State Key Laboratory of Experimental Hematology, Haihe Laboratory of Cell Ecosystem, Institute of Hematology and Blood Diseases Hospital, Chinese Academy of Medical Sciences & Peking Union Medical College, Tianjin, 300020 China; 2 National Clinical Research Center for Blood Diseases, Institute of Hematology and Blood Diseases Hospital, Chinese Academy of Medical Sciences & Peking Union Medical College, Tianjin, 300020 China; 3 Tianjin Key Laboratory of Cell Therapy for Blood Diseases, Institute of Hematology and Blood Diseases Hospital, Chinese Academy of Medical Sciences & Peking Union Medical College, Tianjin, 300020 China; 4 Department of Hematology, The Affiliated Hospital of Qingdao University, Qingdao, 266000 Shandong China; 5 Juventas Cell Therapy Ltd, Tianjin, 300384 China

**Keywords:** CD22, HIB22, CD19, CAR-T, Sequential therapy, B-ALL, Antigen escape

## Abstract

**Background:**

CD19 chimeric antigen receptor (CAR) therapy has achieved impressive success in relapsed or refractory (R/R) B-cell malignancies, but relapse due to antigen escape is increasingly appearing reported. As the expression profile of CD22 is similar to that of CD19, CD22 has become a candidate target when CD19 CAR-T therapy fails.

**Methods:**

A novel CD22 CAR incorporating scFv derived from an HIB22 hybridoma which bound the first and second Ig-like extracellular domains of CD22 antigen was constructed. Preclinical investigation of the CD22 CAR-T therapy against B-cell malignancies was evaluated by coculturing CD22 CAR-T cells with tumor cell lines or primary blasts from patients in vitro and using a xenograft mouse model in vivo. Further clinical study of CD22/CD19 CAR-T sequential therapy was conducted in 4 R/R adult B-cell acute lymphoblastic leukemia (B-ALL) patients.

**Results:**

The novel CD22 CAR-T treatment had specific cytotoxicity to CD22 + target cells, and the survival time of mice in the CD22 CAR-T treatment group was significantly prolonged. Furthermore, it’s validated that sequential CD22/CD19 CAR-T therapy was significantly superior than single CD19 or CD22 CAR-T treatment in a relapse xenograft model. All 4 patients achieved complete remission (CR) with negative minimal residual disease (MRD), including 3 patients who had received prior CD19-related immunotherapy. The proliferation of CD19 and CD22 CAR-T cells was observed respectively in vivo, and 3 of the 4 patients experienced cytokine release syndrome (CRS); 2 of these patients had grade 1 CRS and 1 had grade 3 CRS. Long term follow-up showed that 3 of the 4 (75%) patients had sustained CR for up to 1 year. Analysis of antigen expression in the relapsed patients demonstrated that loss or diminution of CD19 and CD22 expression might cause antigen escape from CAR-T surveillance.

**Conclusions:**

In summary, the novel CD22 CAR-T therapy was validated with antitumor effects both in vitro and in vivo. Furthermore, our study demonstrated the safety and robust efficacy of sequential CD22/CD19 CAR-T therapy in xenograft models and clinical trials, especially as the salvage treatment for R/R B-ALL patients with antigen loss or in whom anti-CD19 related immunotherapy failure failed.

*Trial registration:* Chinese Clinical Trial Registry (ChiCTR): ChiCTR1900025419, Supplementarily registered 26 August, 2019.

**Supplementary Information:**

The online version contains supplementary material available at 10.1186/s40164-022-00270-5.

## Background

Cancer immunotherapy based on chimeric antigen receptor CAR-T represents a new era for curing multiple tumors, especially hematological malignancies [1]. Extensive application of CD19 CAR-T therapy has resulted in complete remission (CR) in more than 80% of B-ALL patients [2–4]. Although anti-CD19 CAR therapy has shown great success, relapses associated with antigen escape have been increasingly linked to treatment failure, accounting for 10–65% of relapses in pediatric patients with relapsed/refractory (R/R) B-ALL in multiple clinical trials [4–7]. A variety of possible escape mechanisms, including alternative splicing of CD19, frameshift mutations and leukemia lineage transformation, have been reported to limit the therapeutic effect of CD19-related immunotherapy [8–10].

According to preclinical studies, combining antigen strategies in CAR-T therapy can work synergistically to overcome interpatient variability in antigen expression [11, 12], reduce disease recurrence mediated by antigen downregulation or loss and improve the efficacy of CAR-T therapy. CD22 is highly expressed in most B-cell malignancies and is expressed only on B cells [13]. Many clinical trials have investigated the efficacy of immunotherapy targeting CD22 [14–16]. Epratuzumab is a CD22 monoclonal antibody with a certain effect in adults and children with B-ALL [17–19]. CD22 immunotoxin has a certain therapeutic effect on hairy cell leukemia and B-ALL [20, 21]. CD19/CD22 bispecific, cocktail and sequential CAR-T therapies exhibit great efficacy in B-ALL patients, especially for patients with relapse after CD19-related immunotherapy [22–24].

In this study, novel anti-human CD22 CAR was constructed that recognized the first and second Ig-like domains of CD22. The CD22 CAR-T cells showed efficient killing activity in vitro and prolonged survival in a B-cell lymphoma xenograft murine model. After infusion of CD22 and CD19 CAR-T cells, all of 4 patients with R/R B-ALL achieved minimal residual diseade (MRD)-negative CR, which demonstrated the safety and efficiency of sequentially combined CD22 CAR-T and CD19 CAR-T cell therapy.

## Methods

### Antibody homology modeling

The “create homology models” module of the Discovery Studio (DS) software was used to construct HIB22 a homology model of HIB22. After searching the amino acid sequence of HIB22 in the protein data bank (PDB) structure database, one hundred of best matched templates were ranked on the basis of the E-value. To perform further modeling, five templates with lower E-values, higher resolutions and longer alignment lengths were selected. Approximately 100 models were built for HIB22 scFv and the final model was determined by parameters given of the DS software as previously described [25].

### Antibody-antigen docking

The ZDOCK and RDOCK modules in DS were used to construct the bound complex between human CD22 (hCD22) and HIB22. The crystal structure of hCD22 is available in the PDB database (PDB ID: 5VKJ). HIB22 scFv was set as the binding receptor and the binding region of hCD22 was restricted to domains 1 and 2, according to previous data [26]. The docking algorithm produced 54,000 binding poses. Qualified poses were choosen based on principles described before for RDOCK refinement. The CHARMm Polar H forcefield was automatically added during this process. Analyze The RDOCK results were analyzed as previous described [25]. The ultimate binding model was determined after comprehensive consideration of receptor-ligand binding interface analysis and RDOCK scores.

### CAR lentiviral vector production and T cell transduction

The anti-human CD22 scFv and anti-human CD19 scFv were derived from hybridoma clone HIB22 and HI19a respectively, established at the Institute of Hematology & Blood Diseases Hospital, Chinese Academy of Medical Sciences & Peking Union Medical College (CAMS & PUMC). Anti-human CD22 scFv or anti-human CD19 scFv was linked with the CD8α signal peptide, CD8α transmembrane domain, 4-1BB costimulatory and CD3ζ cytoplasmic region and assembled into lentiviral pCDH plasmids with GFP as the tag protein. The lentiviruses were produced by transfecting HEK293T cells with CAR plasmids and packaging plasmids including PMD2.g (Invitrogen, USA), pMDLg/pRRE and pRSV-Rev (Biovector Science Lab, China). After isolation from the peripheral blood of healthy donors, T cells were cocultured with CD3/CD28 human T-activator Dynabeads (1 × 10^6^/ml) (Gibco, USA) and recombinant human IL-2 (100–200 U/ml) (R&D, USA) in lymphocyte serum-free medium KBM581 (Corning, USA). T cells were transduced with lentiviruses for 24 h and incubated in medium changed every 2 days, as previously described [27, 28].

### Cell lines and patient samples

Four kinds of target cell lines, including the Burkitt lymphoma cell lines Namalwa, Daudi and the leukemia cell lines Nalm-6 and K562, were cultured in RPMI-1640 medium with 10% FBS (Gibco, USA). HEK293T cells were cultured in DMEM medium (Gibco, USA) with 10% FBS. Peripheral blood mononuclear cells (PBMCs) and bone marrow mononuclear cells (BMMCs) were obtained from B-ALL patients admitted to the Institute of Hematology & Blood Diseases Hospital, CAMS & PUMC, with written informed consent provided by all subjects. IMDM medium was utilized to culture primary BMMCs cells with 15% FBS, 100 ng/ml rhFLT3-L, 100 ng/ml rhSCF and 50 ng/ml rhTPO (PeproTech, USA).

### CRISPR/Cas9 Editing of cell lines

According to the GeCKO human sgRNAs library, specific guide-RNAs were designed and cloned into the LentiCRISPR v2 plasmid (Addgene Plasmid 52,961), and then transformed into the JM109 bacteria. The packaging plasmids PMD2.g (Invitrogen, USA), pMDLg/pRRE and pRSV-Rev (Biovector Science Lab, China) were co-transfected into HEK293T cells. CRISPR supernatants were collected after 2 days, and filtered through a 0.45 μm low protein binding membrane (Millipore, Billerica, Massachusetts, USA). The supernatants were then concentrated by means of high-speed centrifugation, resuspended in PBS, and utilized immediately or kept at − 80 °C. The process of viral transduction employed the use of 1 × 10^5^ Namalwa cells which were then incubated with 10 μl of concentrated viral supernatant for 2 days, followed by expansion in RPMI with 10% FBS (Gibco, USA) and the assessment via flow cytometry, then the cells were sorted with phenotypic changes and single-cell cloning. To ensure a frameshift mutation in the CD19 or CD22 locus, sequencing was carried out on single-cell clones in order to validate genotypic changes via PCR.

### Cytotoxicity assay of CAR-T cells

Among the 4 kinds of target cell lines, the Namalwa, Daudi and Nalm-6 cells were CD22 positive, while the K562 cells were CD22 negative and were used as negative control target cells. When T cells were cocultured with target cell lines or patient BMMCs, the effector to target (E:T) ratios of the coculture system were 1:8, 1:4, 1:1, 4:1 and 8:1 in 24-well plates. After coculture for 24 or 48 h, the percentage of residual target cells was detected via flow cytometry.

### Determination of CD107a positive cells

T cells (1 × 10^5^) were mixed with target cells or patient BMMCs in 200 μl medium in triplicate with addition of IL-2 (50U/ml) and anti-human CD107a antibody. After coculture for 1 h, the ion blocking drug monensin (1 μM, Sigma) was added to the coculture medium. After 4 h, the cells were collected and the proportion of CD107a-positive T cells was analyzed via flow cytometry.

### Assays of cytokine release level

Blood samples were collected from peripheral blood and centrifuged for the serum stored at − 80 °C before analysis. Cytokines were measured using BD Cytometric Bead Array (CBA) human Th1/Th2/Th17 Cytokine Kit (BD bioscience. USA) and ELISA kits for mouse or human interferon gamma (IFN‐γ), tumor necrosis factor (TNF‐α), interleukin-2 (IL-2), IL-6, (R&D systems.USA) as per the manufacturer’s instructions.

### Flow cytometry

Flow cytometry assays were performed on an LSRII or FACSCantoII (BD Bioscience, USA) and analyzed by FlowJo 7.6.1 software. The percentage of CAR-T cells was determined by staining with goat anti-mouse IgG, F(ab')_2_ fragment specific antibody labeled with biotin (Jackson Immunoresearch, USA) for the detection of both GFP- and F(ab’)- positive cells. The following antibodies used in this study were purchased from Biolegend, USA, including CD22 (clone HI22), CD19 (clone HI19), CD3 (clone HIT3a), CD33 (clone WM53), CD34 (clone 561) and CD107a (clone H4A3).

### In vivo murine studies

Female NOD/SCID mice at the age of 6–8 weeks were irradiated at 1.5 Gy. After irradiation, the lymphoma cell line Namalwa was intravenously inoculated into mice at day 0 to establish the lymphoma xenograft mouse model. T cells were intravenously administered to mice on day 4 and 5. Mice in the sequential treatment group received 6 × 10^6^ sorted CD22 CAR-T (infection efficiency 50–60%) cells on day 4 and 6 × 10^6^ sorted CD19 CAR-T (infection efficiency 50–60%) cells on day 5. Mice in the CD19 CAR-T group received 6 × 10^6^ sorted CD19 CAR-T cells on day 4 and day 5, respectively. While, mice in the CD22 CAR-T group received 6 × 10^6^ sorted CD22 CAR-T on day 4 and day 5, respectively. Then, the body weight was monitored and the survival time was measured from the date of inoculation to death. To test the infiltration of tumor cells, samples from the bone marrow, spleen, and liver were dissected and analyzed by pathological examination.

### Clinical protocol design and evaluation of toxic effects

The clinical trial (ChiCTR1900025419) for the evaluation of CAR-T treatment in relapse/refractory (R/R) hematological malignancies was conducted at the Institute of Hematology & Blood Diseases Hospital, CAMS & PUMC. From July 2018 to June 2019, 4 adult patients with R/R B-ALL were enrolled in the clinical trial to evaluate the clinical efficacy of sequential CD22 CAR-T and CD19 CAR-T therapy. Both CD19 and CD22 expression were detected in all 4 patients. End points for this study are safety and efficacy of sequential CD22 CAR-T and CD19 CAR-T therapy.

The patients underwent lymphodepletion chemotherapy including fludarabine (30 mg/m^2^/day from day -4 to -1) and cyclophosphamide (350 mg/m^2^/day on day -4 and day -3), followed by sequential infusion of 1 × 10^6^/kg CD22 CAR-T cells and 1 × 10^6^/kg CD19 CAR-T cells on days 1 and 2, respectively. To evaluate the CAR-T therapy response, BM and PB samples were collected on days 14 and 28. CR and relapse status were assessed according to National Comprehensive Cancer Network (NCCN) guidelines, version 1.2016. The definition of MRD negativity was less than 0.01% blasts in BM detected via flow cytometry. The grade of CRS was in accordance with Lee’s grading system [29].

### Persistence and subtype of CAR-T cells

PBMCs and BMMCs were collected before infusion and multiple time points during immunotherapy. The proportion of CAR-T cells in circulating T cells was detected by flow cytometry. Copy numbers of both CD19 and CD22 CARs in genomic DNA were assayed separately by real-time quantitative polymerase chain reaction (RT-qPCR) as described in the Additional file 1.

Peripheral T cells in circulation were subtyped into naive T cells (T_N_), central memory T cells (T_CM_), effector memory T cells (T_EM_), and effector T (T_E_) cells by flow cytometry analysis.

### Statistical analysis

At least 3 biological replicates were performed, and the results are reported as the mean ± SEM. Statistics were analyzed by Student’s t test, Kaplan–Meier methods and a log-rank test via GraphPad Prism 7. P values < 0.05 were considered statistically significant. Generally, the p values were defined as *p < 0.05, **p < 0.01, ***p < 0.001 and ****p < 0.0001 in statistics.

## Results

### Construction of a novel CD22 CAR-T

**Fig. 1:**
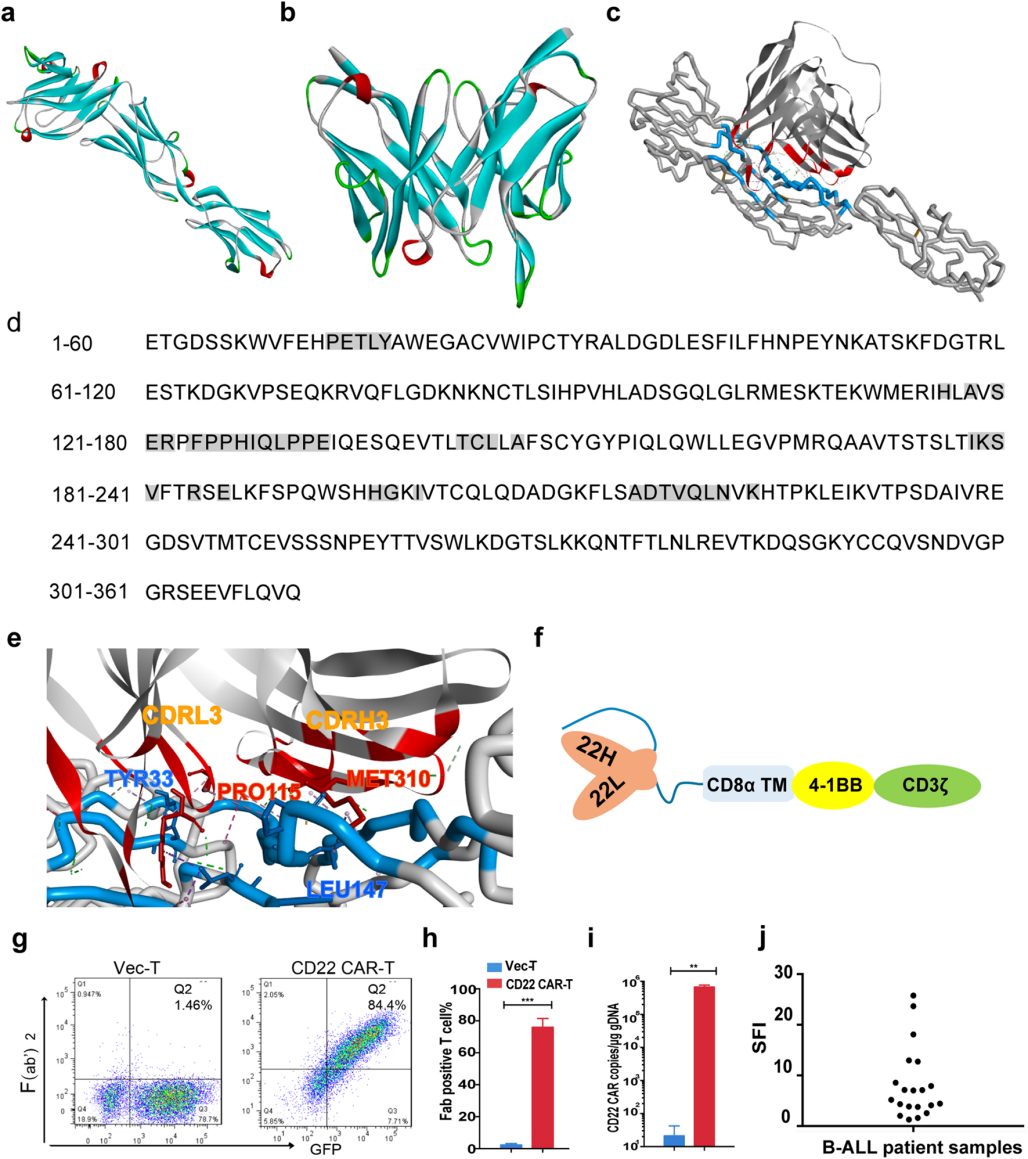
Construction and structural characteristics of a novel CD22 CAR derived from HIB22.** a** Homology models of hCD22 (ECD, domains 1–3). **b** Homology models of HIB22 scFv. **c** Docking mode of HIB22 scFv with CD22(ECD, domains 1–3). Blue, hCD22 ECD epitopes; Red, HIB22 epitopes. **d** Sequence of hCD22 (ECD, domains 1–3). Gray background, amino acids as epitopes predicted involved in binding with hCD22 (ECD, domains 1–3). **e** Partial interaction mode between HIB22 scFv and hCD22 (ECD, domains 1–3). Blue, hCD22 (ECD, domains 1–3) epitopes and presentative amino acids involved in non-bond interaction. Red, HIB22 scFv epitopes and presentative amino acids involved in non-bond interaction. **f** Schematic of CD22 CAR construct. CD8α TM, CD8α transmembrane domain. CD3**ζ**, CD3 zeta domain. **g** Expression of CAR on T cells surface. The CD22 CARs or empty vectors were transduced to T cells to obtain corresponding CD22 CAR-T cells or control VEC-T cells. CAR expression was analyzed by FACS. **h**, **i** Collective analysis of expression of CAR **h** and copies number of CAR transduced to genomic DNA **i** from 3 donors. **j** SFI (specific fluorescence index) of CD22 expression on B-ALL patient samples. The SFI is calculated as follows: SFI = (MFI of specific antibody—MFI of isotype control)/MFI of isotype control, where MFI is the mean fluorescence intensity

In this study, a CAR targeting the B cell antigen CD22 was constructed from a hybridoma cell line HIB22, which was established at our institute. Epitope mapping of antibody derived from HIB22 hybridoma was done in previous study, by experiments expressing truncated forms of hCD22 cDNA that lack different Ig-domains, which verified that HIB22 derived monoclonal antibody recognized epitopes on the first and the second Ig-like domains of hCD22 [26, 30]. To further identify the docking mode of HIB22 scFv and hCD22, homology modeling and antibody-antigen docking were performed. The crystal structure of hCD22 was available in the PDB database (PDB ID: 5VKJ), and the structure of HIB22 scFv was evaluated by molecular modeling (Fig. 1a, b). The binding mode of hCD22 and HIB22 is shown in Fig. 1c, with amino acids of hCD22 listed as crucial antigen epitopes (Fig. 1d). Alanine scanning mutagenesis demonstrated that the main docking site on HIB22 scFv was the third complementarity-determining region loop in the light chain (CDRL3) and heavy chain (CDRH3), respectively (Fig. 1e, Additional file 2: Table S1, S2). The results above elucidated the docking mode and recognition epitopes between HIB22 scFv and hCD22, which, to the best of our knowledge, is different from existing anti-human CD22 scFvs incorporated into CARs [16, 31–33]. The HIB22 scFv was constructed into a novel CD22 CAR combining the CD8α transmembrane domain, 4-1BB and CD3ζ cytoplasmic signaling domain (Fig. 1f). T cells were loaded with CD22 CAR via lentivirus infection with an infection efficiency greater than 70% and a copies number of CD22 CAR up to 10^5^/μg genomic DNA(Fig. 1g–i).

The B lineage restricted expression profile of CD22 ensured that the side effects caused by off-target of CD22 CAR therapy were reduced. The expression of CD22 on B-cell precursor ALL or lymphoma cell lines (Additional file 2: Fig. S1a) was validated via flow cytometry. BMMC samples derived from B-ALL patients, were evaluated for the expression of CD22 and CD19. As shown in Fig. 1h and Additional file 2: Fig. S1b, the specific fluorescence index (SFI) of CD22 and CD19 on primary blasts demonstrated an interpatient variability among the B-ALL patients. Thus CD22 CAR-T cells are feasible for B-cell malignancy treatment.

### Efficacy of the CD22 CAR-T therapy against B-cell malignant cells from both cell lines and primary B-ALL patients in vitro

**Fig. 2:**
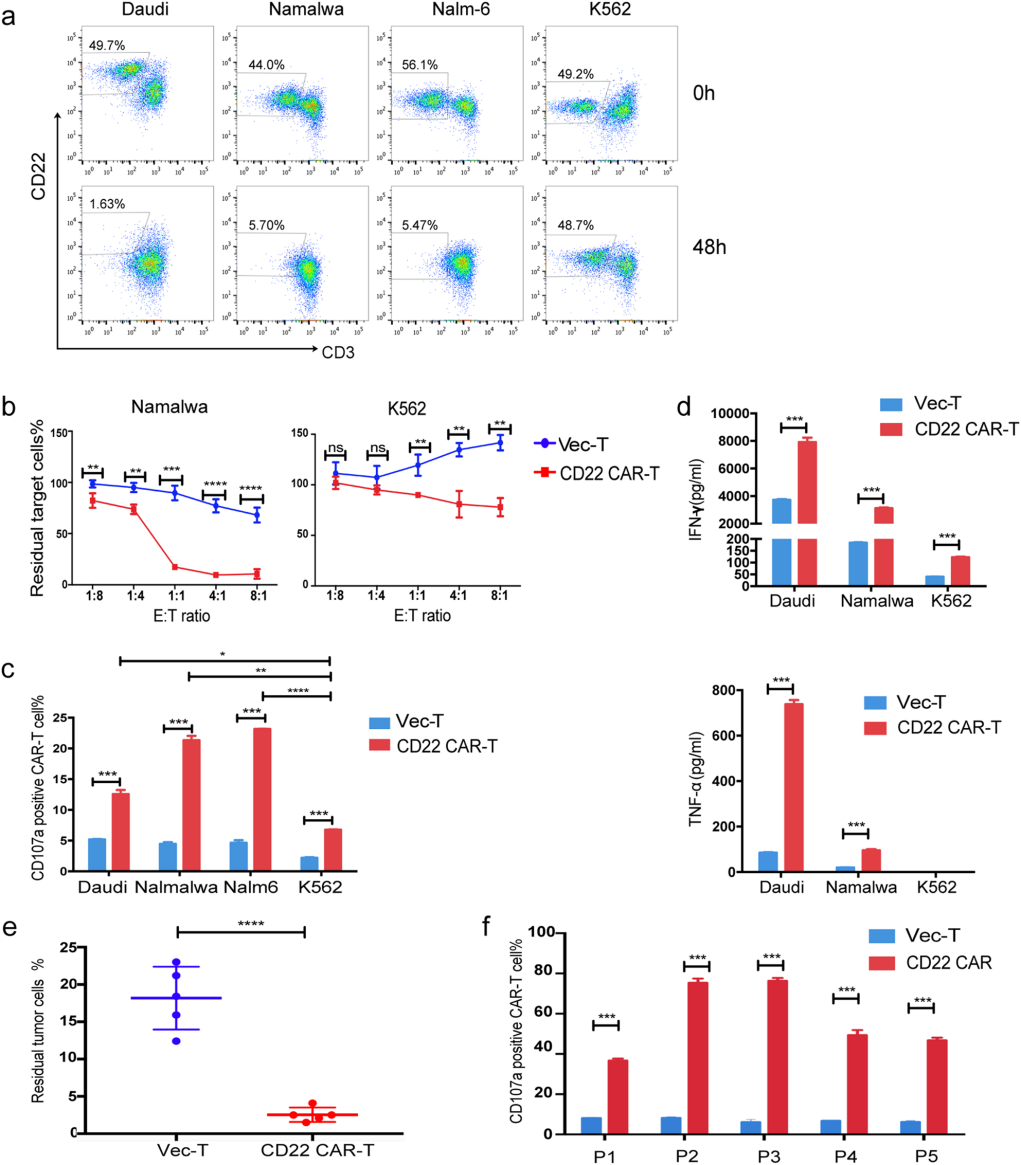
In vitro evaluation of CD22 CAR T cells. **a** A representative diagram of flow cytometry analysis of residual Daudi, Namalwa, Nalm-6 and K562 cells when cocultured with CAR-T cells at 0 h (upper panel) and 48 h (lower panel). **b** Percentage of residual Namalwa or K562 cells detected by flow cytometry after co-culturing with CAR T or VEC-T cells for 24 h at indicated E:T ratio (1:8, 1:4,1:1,4:1,8:1). **c**. Percentage of degranulated CAR-T cells (CD107a positive T cells/CAR positive T cells) after cocultured with Daudi, Namalwa, Nalm-6 and K562 for 24 h. **d** ELISA detection of IFN-γ and TNF-α in the supernatants of VEC-T or CAR-T cocultured with Daudi, Namalwa and K562 for 24 h. **e** Primary ALL cells were collected from 5 patients, and cocultured with CD22 CAR-T cells or vector-T cells at E:T ratio of 1:1 respectively for 48 h. The residual tumor ALL cells was defined as 7AAD^−^CD3^−^GFP^−^CD19^+^CD10^+^ by flow cytometry. **f** Percentage of degranulated CAR-T cells (CD107a positive T cells/CAR positive T cells) after cocultured with patient samples for 5 h

We first evaluated the in vitro effect of CD22 CAR-T therapy. ALL or lymphoma cell lines, Daudi, Namalwa, Nalm-6 and the CD22 negative control cell line K562, were cocultured with CD22 CAR-T cells at an E:T ratio of 1:1. After 48 h, the cells were harvested and tested by flow cytometry. CD22^+^ malignant cell lines were effectively killed, while K562 cells were rarely killed (Fig. 2a). Even if we increased the E: T ratio from 1:8 to 8:1, the cytotoxicity of the CAR-T group on K562 cells was only slightly higher than that of the vector-T group (p = 0.004 at E:T = 8:1) (Fig. 2b). The results demonstrated that the CD22 CAR-T cells had specific cytotoxicity against CD22^+^ target cells.

During coculture of CAR-T cells with ALL or lymphoma cell lines, degranulation and cytokine release of T cells were also detected. After coculture with CD22-positive target cells, the degranulation level of CD22 CAR-T cells, which was marked by CD107a expression, was significantly increased compared with that of vector-T cells. However, CAR-T cells showed a significant lower level of degranulation when cocultured with CD22-negative K562 cells than when cocultured with CD22-positive cells (Fig. 2c). Additionally, the levels of IFN-γ and TNF-α in the coculture supernatant of CAR-T cells with CD22-positive target cells were also significantly increased compared with those of the vector-T cells (Fig. 2d). This result indicated that CD22 CAR-T cells were significantly activated by CD22 positive tumor cells to secrete proinflammatory cytokines and release perforin and granzyme, thereby killing tumor cells.

Next, the cytotoxic effect of CAR-T cells on BMMCs derived from primary ALL patients was examined. BMMCs were collected from 5 ALL patients with variable CD22 expression intensity (Additional file 2: Fig. S1c), and cocultured with T cells. CD22 CAR-T cells showed striking killing activity on all 5 ALL patient samples (Fig. 2e), even for samples with low expression of CD22. In addition, CAR-T cells displayed a significantly high level of activation via degranulation assays (Fig. 2f).

### Antitumor effect of CD22 CAR-T and sequential CD22/19 CAR-T therapy in vivo

**Fig. 3:**
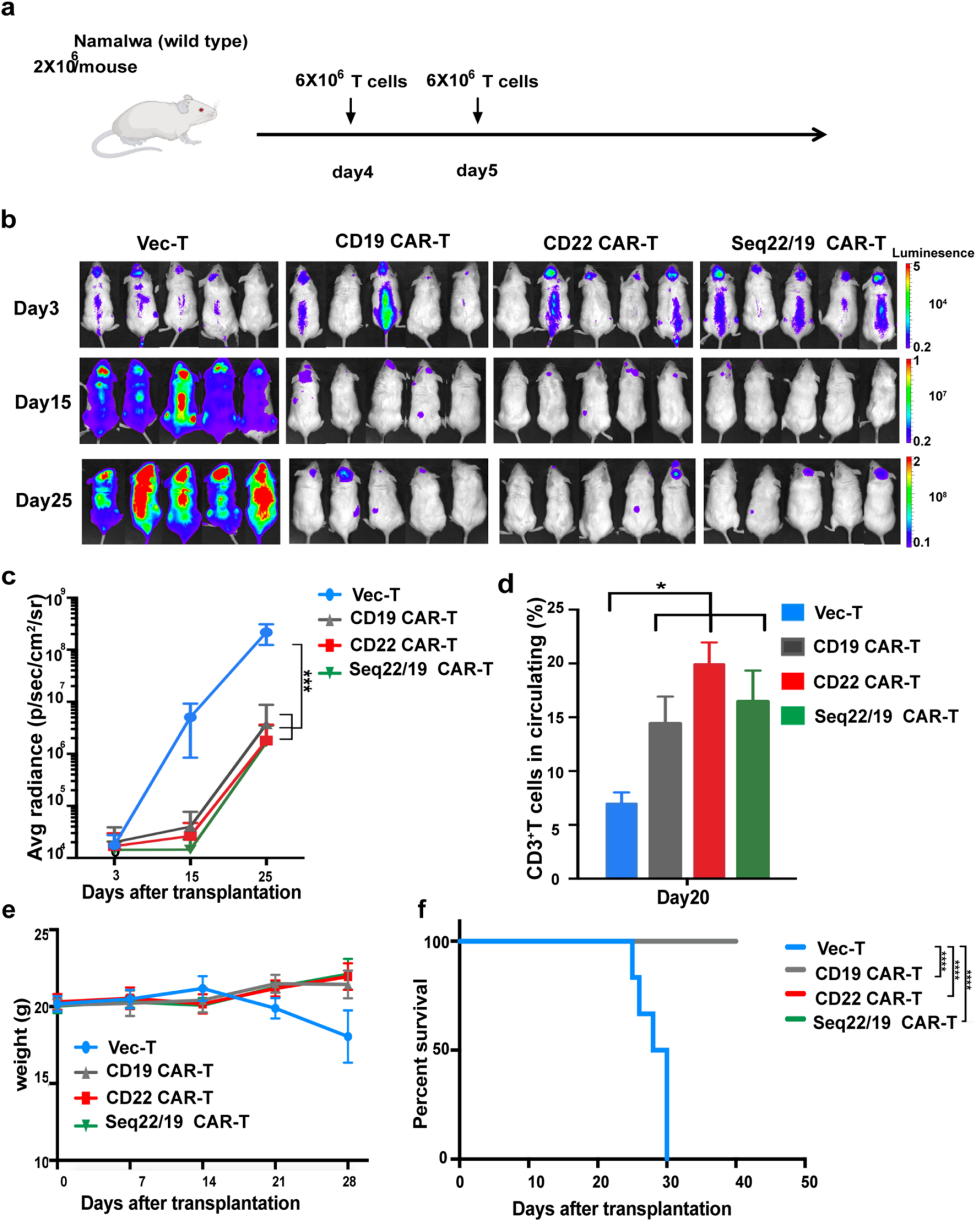
In vivo validation of CD22 CAR-T and CD22/CD19 sequential CAR-T cells in wild-type Namalwa inoculated mice model. **a** Schematic of in vivo evaluation of CAR-T. NOD/SCID mice were challenged with 2 × 10^6^ wild type Namalwa cells on day 0, Mice in the sequential treatment group received 6 × 10^6^ CD22 CAR-T(infection efficiency 50%–60%) cells on day 4 and 6 × 10^6^ CD19 CAR-T (infection efficiency 50%–60%) cells on day 5. Mice in the CD19 CAR-T group received 6 × 10^6^ CD19 CAR-T cells on day 4 and and day 5, respectively. Mice in the CD22 CAR-T group received 6 × 10^6^ CD22 CAR-Tcells on day 4 and day 5, respectively. b. IVIS imaging of disease burden monitored by BLI at the indicated time points. **c**. Average radiance quantification (p/sec/cm^2^/sr) for Namalwa at the indicated time points. **d** T cell persistence in peripheral blood on day 20. (n = 5 per column) **e** Average body weight of two groups after CAR-T cell treatment. **f** Kaplan–Meier survival curves of VEC-T and CAR-T treatment groups. The *P*-values were determined by log-rank test. *P* < 0.001 when group Vector-T compared with CAR-T. N = 5–6 for VEC-T or CAR-T group

To further study the efficacy of CD22 CAR-T and sequential CD22/19 CAR-T therapy in vivo, we established a CD19 and CD22 both positive lymphoma xenograft mouse model by intravenously inoculating the lymphoma cell line Namalwa cells into NOD/SCID mice. Pathological examination confirmed the tumor cells infiltration in the bone marrow, kidney and other organs of affected mice, accompanied by venous congestion and inflammatory cell infiltration (Additional file 2: Fig. S2a). We explored the efficiency of both CD22 CAR-T and CD19/CD22 sequential CAR-T therapy in vivo for the sake of overcoming CD19 and CD22 expression variability and preventing relapse associated with antigen escape. CD19 CAR with 4-1BB costimulatory domain (Additional file 2: Fig. S2b) used in this study was derived from HI19a hybridoma and has been shown to have therapeutic benefits in preclinical and clinical studies [25, 27]. CAR-T cells or vector-T cells were sequentially administered on days 4 and 5 after inoculation of CD19^+^/CD22^+^ Namalwa cells (Fig. 3a). Then, the tumor burden in the mice was examined by bioluminescence imaging (BLI). CD22 CAR-T and sequential CD22/CD19 CAR-T groups showed relatively similar therapeutic effects against the growth of Namalwa cells in vivo (Fig. 3b, c). Mice that received CAR-T had significantly higher levels of peripheral T cells and circulating cytokins including human INF-γ, TNF-α and IL-6 (Fig. 3d and Additional file 2: Fig. S2c). Meanwhile, no loss of body weight was found in the CAR-T treatment groups (Fig. 3e), indicating that all three kinds of CAR-T treatment regimens were safe. Compared with the vector-T group, CD22 CAR-T and sequential CD22/19 CAR-T significantly prolonged the survival time of mice (Fig. 3f), demonstrating that both CD22 CAR-T and sequential CD22/CD19 CAR-T were safe and efficacious.

**Fig. 4:**
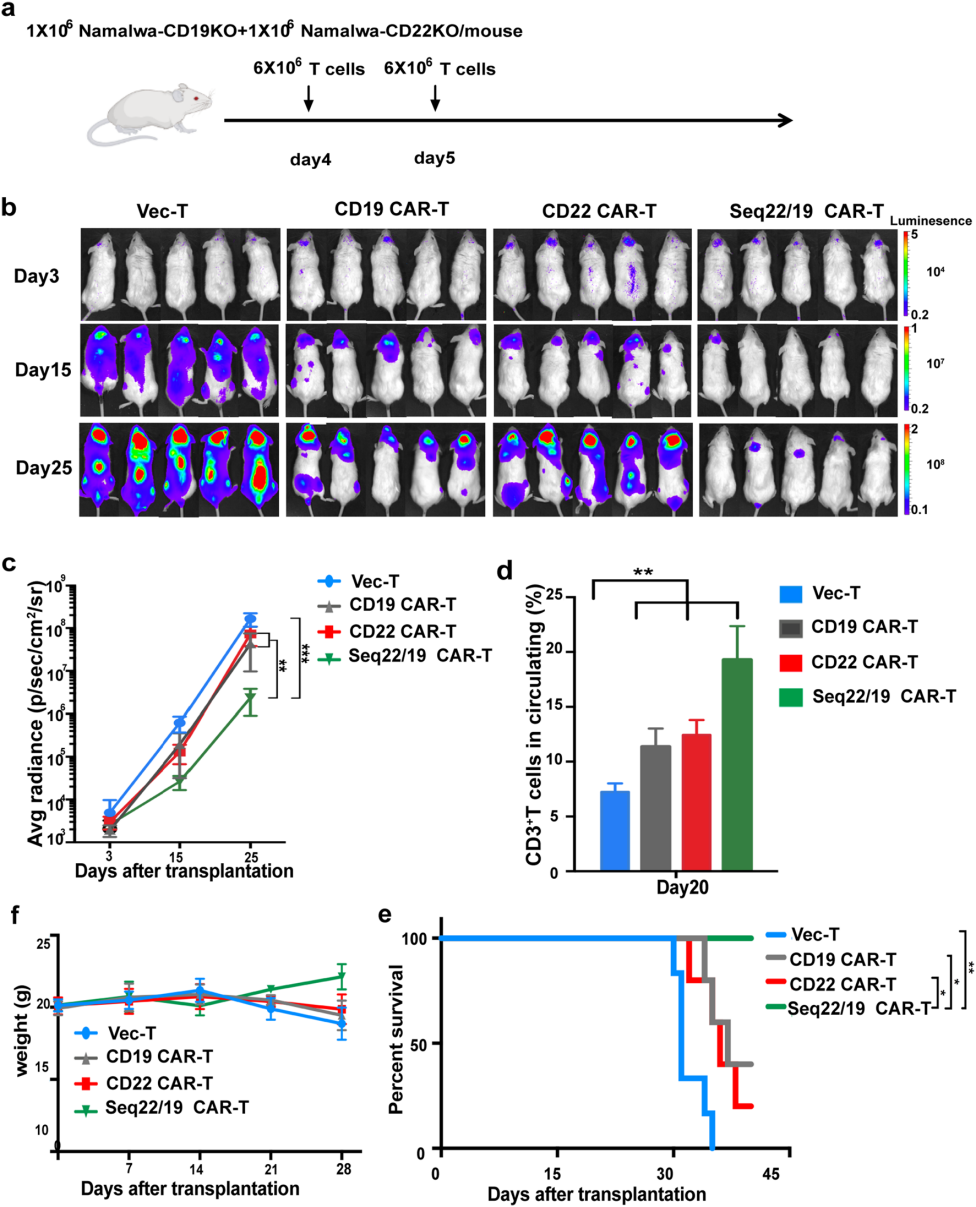
In vivo validation of CD22 CAR-T and CD22/CD19 sequential CAR-T cells in Namalwa-CD19KO and CD22KO inoculated mice model. **a** Schematic of in vivo evaluation of CAR-T. NOD/SCID mice were challenged with a mixture of 1 × 10^6^ Namalwa-CD19KO, and 1 × 10^6^ Namalwa-CD22KO lines on day 0, Mice in the sequential treatment group received 6 × 10^6^ CD22 CAR-T(infection efficiency 50%–60%) cells on day 4 and 6 × 10^6^ CD19 CAR-T (infection efficiency 50%–60%) cells on day 5. Mice in the CD19 CAR-T group received 6 × 10^6^ CD19 CAR-T cells on day 4 and and day 5, respectively. Mice in the CD22 CAR-T group received 6 × 10^6^ CD22 CAR-Tcells on day 4 and day 5, respectively. **b** IVIS imaging of disease burden monitored by BLI at the indicated time points. **c** Average radiance quantification (p/sec/cm^2^/sr) for Namalwa at the indicated time points. **d** T cell persistence in peripheral blood of mice on day 20. (n = 5 per column) **e** Average body weight of two groups after CAR-T cell treatment. **f** Kaplan–Meier survival curves of VEC-T and CAR-T treatment groups. The *P*-values were determined by log-rank test. *P* < 0.001 when group Vector-T compared with CAR-T. N = 6 for VEC-T or CAR-T group

To simulate the dilution or even loss of CD19 and CD22 antigen seen at clinical stage, we constructed CD19KO (CD19 knock out) or CD22KO (CD22 knock out) Namalwa cell lines through CRISPR-Cas9 technology (Additional file 2: Fig. S2d). Mice were injected with a 1:1 mixture of Namalwa-CD19KO and Namalwa-CD22KO cells (Fig. 4a). Analysis of BLI revealed that sequential administration of CD22/CD19 CAR-T eradicated Namalwa cells significantly while both CD19 and CD22 single CAR-T cells only partially reduced disease burden (Fig. 4b, c). These differences were also reflected in the expansion of T cells and circulating cytokines in vivo (Fig. 4d and Additional file 2: Fig. S2e). Sequential administration of CD22 and CD19 CAR-T was safe and could prolong the survival of mice more effectively compared to infusion of single CAR-T cells (Fig. 4e, f).

All of the above results illustrated that the application of the novel CD22 CAR-T therapy was an effective medical cure for CD22^+^ B-cell malignancy in vivo. Besides, sequential CD22/CD19 therapy exhibited identical efficiency in parental Namalwa inoculated model and showed signicant higher activity than single CAR-T treatment in the antigen loss murine model.

### Patient characteristics, clinical responses and long term survival

Four adults with R/R B-ALL were enrolled in this study to receive CD22/CD19 sequential CAR-T therapy. The clinical characteristics of the patients are summarized in Table 1. Among the four patients, the median age was 28 years old (range,18–40). The tumor burdens in patients varied before sequential CAR-T cells infusion. Two patients (patients 1 and 3) had a higher leukemia burden, with 64.93 and 22.54% bone marrow blasts, respectively. The other two patients had minimal residual disease (MRD) detected by flow cytometry. Three patients had previously been treated with CD19-related immunotherapy. Regarding the efficacy of prior CD19-related immunotherapy, patient 1 was refractory to blinatumomab therapy with diminished expression of CD19 and CD22; patient 2 maintained an MRD-positive status after infusion of CD19 CAR-T cells; and patient 3 maintained clearance of blasts for only two weeks and then the disease quickly progressed after blinatumomab therapy.

**Table 1:** Patient characteristics and clinical response

Patient number	Age/ sex	Leukemia type and genetic abnormalities	BM blasts burden	Prior lines of treatment	Prior immunotherapy	Dose of CAR-T cells: CD22 CAR^#^/CD19 CAR^#^	Clinical response
1	26/M	Ph-like ALL (BCR-JAK2 fusion, IKZF1 deletion)	64.93%	4	Blinatumomab	1/1	CR (MRD^−^)
2	18/M	Ph^−_^ ALL (TP53 loss, NRAS mutation)	0.33% (MRD^+^)	4	CD19 CAR-T	1/1	CR (MRD^−^)
3	30/M	Ph^−_^ALL	22.54%	4	Blinatumomab	1/1	CR (MRD^−^)
4	40/M	Ph^+^-ALL (V299L mutaion)	0.19% (MRD^+^)	2	No	1/1	CR (MRD^−^)

*M* male, *Ph* Philadelphia chromosome, Bone marrow blast burden was detected by flow cytometry, ^#^ × 10^6^ cells/kg of body weight

**Fig. 5:**
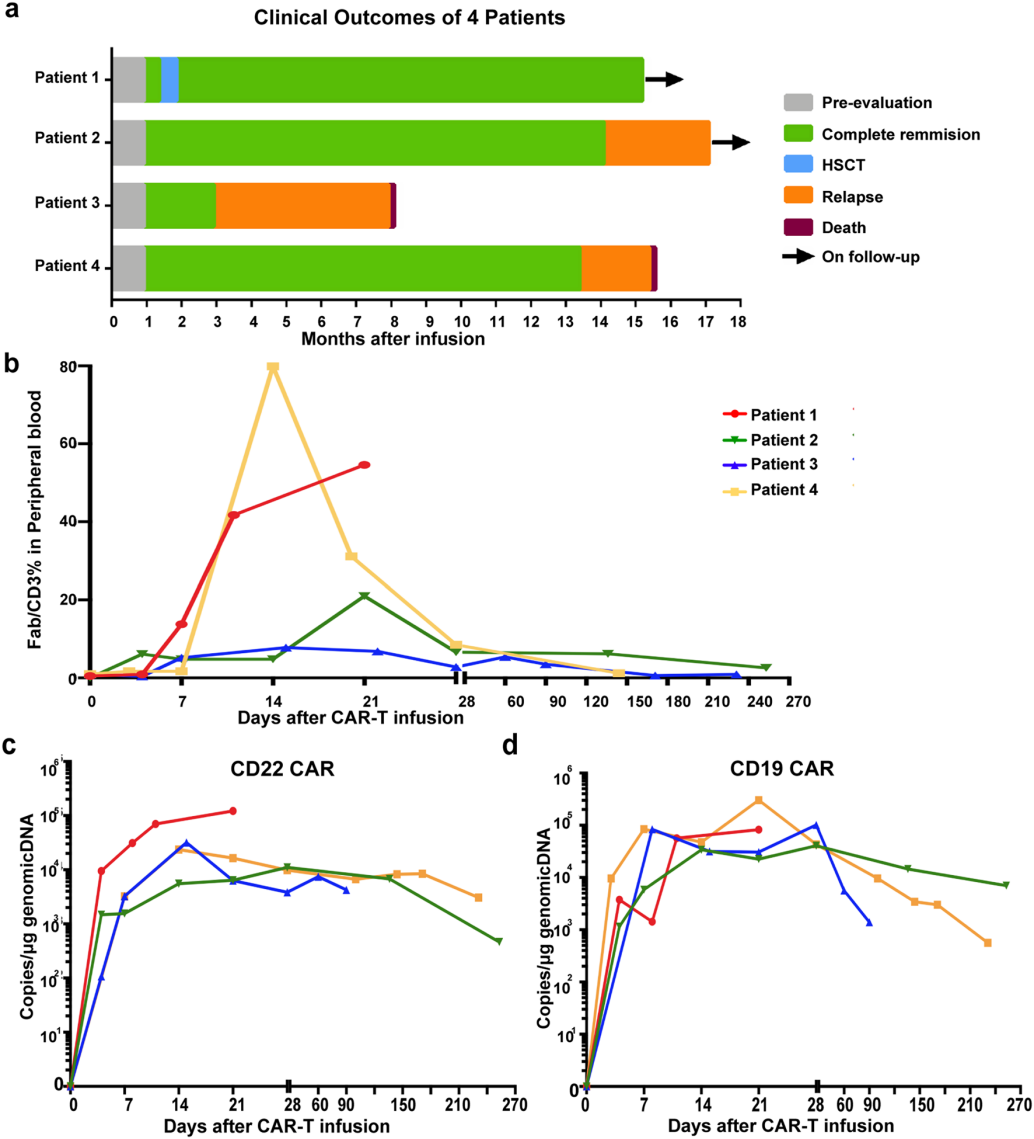
The robust expansion of CAR-T cells and antileukemic activity of sequential CD22/CD19 CAR-T therapy. **a** Response and long term follow-up of each patient who received CD22/CD19 CAR-T sequential therapy. **b** Percentage of circulating CAR-T (Fab positive T) cells in total T cells analyzed by flow cytometry. **c**, **d** Copy numbers of CD22 CAR **C** and CD19 CAR **D** which integrated in genomic DNA per μg PBMC genomic DNA in each patient at different time points

After CD22/CD19 sequential CAR-T therapy, all four patients (100%) achieved MRD negative CR (sensitivity is negative < 0.01%). Flow cytometry assessment indicated that 3 of 4 patients experienced MRD negative CR on day 28, and 1 patient achieved MRD negative CR 90 days after T-cell infusion. The median follow-up in this study was 13 months (range, 8–17 months). Three of four (75%) patients (patients 1, 2 and 4) achieved sustained remission up to 1 year. Patient 1, who was diagnosed with Ph-like ALL, with the BCR-JAK2 fusion gene and IKZF1 deletion (Table 1), experienced MRD-negative CR with a molecular remission on day 28 after T-cell infusion. This patient then underwent allogeneic hematopoietic stem cell transplantation (allo-HSCT) 2 months after sequential CAR-T therapy and remained in an ongoing disease-free survival (> 12 months) state at recent follow-up (Fig. 5a). Patient 2, who received CD19 CAR-T treatment prior to enrollment in this sequential therapy, with genetic lesion of TP53 loss, was in a morphologic CR with an MRD of 0.03% bone marrow blasts on day 28. In a follow-up re-examination two months later, this patient achieved MRD-negative CR and sustained CR up to 1 year. Patient 4, who was diagnosed with Ph^+^ ALL, remained in remission after sequential infusion of CAR-T cells for 1 year. Among the 4 patients who attained CR, 3 patients relapsed after sequential CAR-T cell therapy, with CR durations of 12 months in patient 2, 2 months in patient 3, and 12 months in patient 4 (Fig. 5a). Patients 3 and 4 died due to disease progression 8 and 15 months after T-cell infusion, respectively.

### Kinetics of sequentially infused CAR-T cells at the clinical stage

Flow cytometry and qPCR examination were performed to monitor the kinetics and persistence of peripheral CAR-T cells. Of 4 patients, the amplification of CAR-T cells peaked on days 14–21 after infusion (Fig. 5b). The peak proportion of CAR-T cells in total circulating T cells varied from 7.8 to 79.9% as analyzed via flow cytometry (Fig. 5b). qPCR analysis demonstrated that CD19 and CD22 CAR-T cells both expanded significantly but with a separate expansion pattern during sequential therapy. Three weeks after T-cell infusion in patient 1, CD22 CAR copy numbers increased to a higher level of 10^5^/μg genomic DNA (Fig. 5c), meanwhile, CD19 CAR peaked to a lower degree (Fig. 5d), partially due to stronger expression of CD22 antigen in BM blasts before sequential therapy (Additional file 2: Fig. S3a). We observed roughly the same amplification patterns, with copies ranging from 10^3^ to 10^5^ per μg, between the two kinds of CAR-T cells in the other 3 patients (Fig. 5c, d). Although decreased to a lower level at 1 month after treatment, circulating CAR-T cells sustained for a prolonged follow-up period of 9 months in patient 2 and 4, which resulted in prolonged leukemia free survival for up to 1 year (Fig. 5c, d). Evaluation of T-cell subtypes demonstrated that the proportion of the CD8-positive T subtype significantly increased accompanied by a gradual decrease in CD4^+^ T cells (Additional file 2: Fig. S3b). Flow cytometry analysis of memory and effector cell markers revealed that CD8^+^ T_EM_ and T_E_ cells significantly expanded after infusion of CAR-T cells (Additional file 2: Fig. 3c).

### Toxicities associated with CD22/CD19 sequential CAR-T therapy

**Fig. 6:**
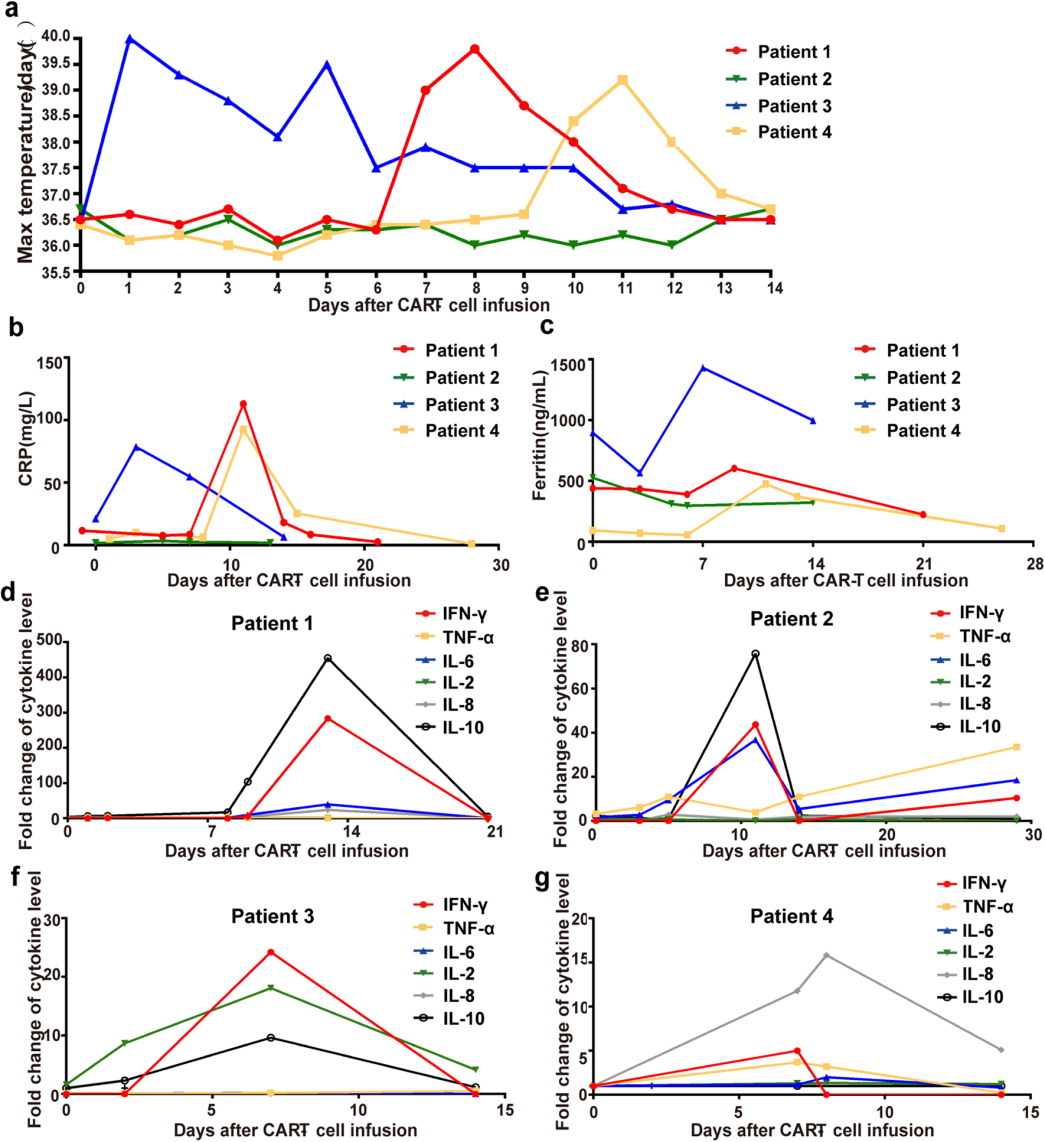
Safety profile of CD22/CD19 CAR-T therapy. **a** Temperature change and occurrence of fever of each patient after CD22/CD19 CAR-T cells infusion. **b**, **c** C-reactive protein (**b**) and ferritin (**c**) in circulating peripheral blood of each patient at different time points. **d**–**g** Change of cytokine levels, including IFN-γ, IL-2R, TNF-α, IL-6, and IL-10 in 4 patients after received sequential CAR-T therapy. Fold change of cytokines was calculated via the formula: Fold change of cytokines = cytokine levels at different times/cytokine levels on day -2 or day 0 before CAR-T cells infusion

Toxicity was summarized in Table 2 and Fig. 6. The primary toxicity that occurred in patients was CRS. In our study, 3 of 4 patients experienced CRS, including 2 patients with grade 1 CRS and 1 patient with grade 3 CRS. The onset of CRS in 3 patients ranged from day 1 to day 10. Patient 1 experienced fever on day 8 and then developed grade 1 CAR-T cell related encephalopathy syndrome (CRES) followed by drowsiness, temporary confusion and dysphasia on day 9 (Fig. 6a and Table 2). Elevated C-reactive protein (CRP), ferritin, INF-γ and IL-10 levels accompanied by grade 3 coagulopathy with hypofibrinogenemia were subsequently observed (Fig. 6b–d). Herein, this patient was diagnosed with grade 3 CRS. After the administration of ruxolitinib, symptoms improved dramatically and laboratory examination results gradually return to normal on day 12. Patient 3 developed fever 1 day after sequential T-cell infusion and the fever resolved on day 8 after administration of antibiotics and low dose dexamethasone (Fig. 6a). Patient 4 experienced fever on day 10 accompanied by elevated CRP, ferritin and proinflammatory cytokines (Fig. 6b, c and g). After administration of IL-6 receptor antagonist tocilizumab, the signs and symptoms of CRS were relieved. INF-γ, IL-10 and IL-6 were the principle cytokines elevated in patients with CRS (Fig. 6d–g). Grade 1–2 vomiting, nausea, hypotension, and musculosleletal pain, as symptoms associated with CRS, were also observed and resolved subsequently (Table 2).

**Table 2:** Grade of adverse events in the patients receiving CAR T cells infusion

Patient no.	1	2	3	4
CRS	3	0	1	1
Neurologic events				
Depressed level of consciousness	1			
Dysphasia	1			
Tremor	1			
Hematologic events				
Thrombocytopenia	4			1
Anemia	3		2	1
Neutropenia	4	1	4	3
Other non-hematologic events				
Diarrhea			1	
Fever	2		2	2
Vomiting				1
Nausea				1
Muscle or bone pain	1			
Infection	3	3	3	3
Hypotension	2	1		1
Elevated aminotransferases				1
Elevated blood bilirubin	2	1		2
Hypokalemia	1	1		
Hypocalcemia	1		1	2
Hypoalbuminemia	2			1
Hypofibrinogenemia	3			
Prolonged APTT	1		1

Hematologic events were common and occurred in all of 4 patients, with 3 patients developing grade 3–4 neutropenia, 1 patient developing grade 3 anemia and grade 4 thrombocytopenia. Of these, grade 3–4 neutropenia persisted with a median duration of 12 days (range 5–15 days). Both high-grade anemia and thrombocytopenia in patient 1 lasted for 9 days. Of note, most patients developed grade 1–2 cytopenia before CAR-T cells infusion owing to the pretreatment and lymphodepletion. B cell aplasia occurred in all patients after infusion of T cells (Additional file 2: Fig. S3d). The reconstitution of B cells was firstly observed in patient 3 on day 90 accompanied by a leukemia relapse. For patient 2 and 4, B cell aplasia persisted for more than 6 months with a persistence of CAR-T cells and a sustained CR, which demonstrating a correlation of B cell aplasia and CR duration (Additional file 2: Fig. S3a and Fig. 5a). Hypogammaglobulinemia was detedcted in 3 patients and warrented immunoglobulin replacement. Other adverse events including hyperbilirubinemia, elevated aminotransferases and electrolyte disturbance were limited and reversible (Table 2).

### Relapse after CD22/CD19 sequential CAR-T therapy

**Fig. 7:**
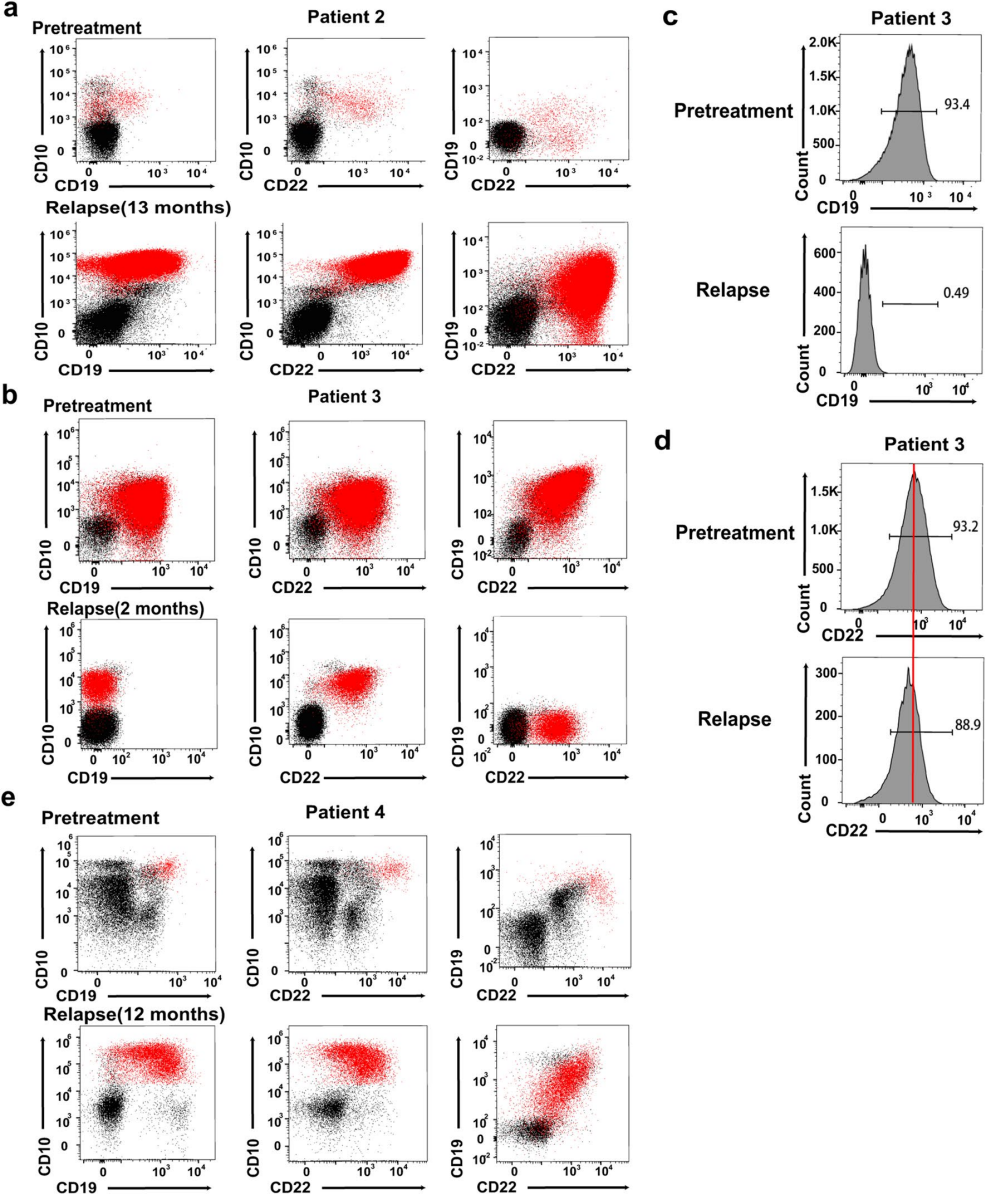
Analysis of CD19 and CD22 cell-surface expression in three patients at pretreatment and relapsed stage. BMMCs from patients before CAR-T cells infusion and relapsed after immunotherapy were obtained, stained with CD45, CD34, CD10, CD33, CD19 and CD22 and analyzed by flow cytometry. Leukemia blasts were gated via CD45/SSC two parameters graph and subgated in CD34 or CD10, followed by analysis of surface expression of CD19 and CD22 on gated cells. Red dots represent leukemia blasts. Black dots represent normal cells. **a**, **b** and **e**. Flow cytometry analysis of BMMCs samples from 3 patients at pretreatment and relapse stage, including patient 4 (**a**), patient 3 (**b**) and patient 2 (**e**). **c.** CD19 expression in patient 3 by histogram analysis at pretreatment and relapse stage indicated loss of CD19 antigen during treatment. **d** Histogram analysis of CD22 on BMMCs samples from patient 3 before CAR-T cells infusion and relapse stage showed CD22 downregulation

To further explore the relapse mechanism after sequential infusion of CAR-T cells, we examined the changes in CD19 and CD22 antigen expression on leukemia blasts by analyzing the MRD of three patient samples obtained at the pretreatment and relapse stages. Patient 2 relapsed 13 months after therapy with leukemia cells expressing both CD19 and CD22 antigens similar to pretreatment (Fig. 7a). Given that the patient achieved clearance of blasts on day 28, the mechanism of CD19- and CD22- positive relapse after a remission of 1 year might partially lie in the limited persistence of CAR-T cells rather than antigen escape.

Patient 3 relapsed 3 months after T-cell infusion despite of detectable levels of circulating CAR-T cells (Fig. 7b). Histogram analysis revealed complete loss of CD19 (Fig. 7c) and downregulation of CD22 (Fig. 7d). Patient 4 relapsed with a diminished expression level of CD19 and CD22 antigen, accompanied by some CD19- and CD22- negative blasts in the bone marrow (Fig. 7e). The results assessed via flow cytometry in these two patients were consistent with those reported preiously [14, 22, 34]. In contrast to the complete absence of CD19 antigen due to gene mutation and selection by immune pressure following immunotherapy, CD22 on reoccurring leukemia cells showed a diminished expression level rather than loss of antigen, which demonstrated a distinctive pattern of acquired resistance to CAR-T cell-mediated surveillance among different antigens.

## Discussion

The impressive efficacy of CD19-directed CAR-T therapy have been validated in numerous clinical trials for pediatric and adult patients with R/R B cell malignancies [2, 25, 35]. However, 40–60% of CR patients relapse within 1 year, and a large proportion of patients are CD19 have diminished or negative relapse [4, 7]. For patients with CD19-positive relapse after CD19-directed immunotherapy, a second infusion of CD19 CAR-T cells has been considered as a possible strategy but CR was achieved in only 21% of B-ALL patients [36]. Under these circumstances, CD22 is increasingly regarded as an effective target for patients with B-cell malignancies, especially relapsed cases with or without CD19 retention after CD19-related immunotherapy [37, 38]. The first phase 1 trial of CD22 CAR-T therapy for R/R B-ALL patients has demonstrated great efficacy in inducing CR in 12/21 patients [14]. Cocktailed or bispecific CD19/CD22 CAR-T therapies have entered the clinical validation phase and have shown robust antileukemic effects [23, 39]. In our study, a novel CD22 CAR derived from an HIB22 hybridoma was constructed and the efficient killing activity both in vitro and in vivo at preclinical stage was observed. We then conducted a CD22 and CD19 sequential CAR-T therapy for 4 adult patients with R/R B-ALL and validated its great clinical efficacy and safety profile.

Several CD22-CARs based on different sources of scFvs have been reported in clinical trials [14, 16, 40]. Previous studies have addressed the crucial role of epitope specificity in CAR efficacy with comparison showing that the m971 scFv, which targets the 5–7 extracellular domain of CD22, seems more efficacious than BL22 scFv, which recognizes extracellular domain 3 [31, 33]. Here, assays of binding to truncated antigens lacking of different Ig domains validated that the CD22 CAR we constructed incorporating HIB22 scFv specifically targeted the first and second Ig-like domains [26]. We further conducted molecular docking and epitope mapping to characterize the HIB22 scFv. To the best of our knowledge, the HIB22 hybridoma derived CD22 CAR is the first CAR that simultaneously targets membrane distal Ig-like 1 and 2 domains of hCD22.

Preclinical function studies of CD22 CAR-T cells with the 4-1BB costimulatory domain were comprehensively evaluated from three aspects, including cytotoxic effects on CD22-positive cell lines, primary samples from patients and B-cell malignancy xenograft models. In vitro validation demonstrated strong activation and cytotoxicity of CD22 CAR-T cells by monitoring residual CD22^+^ tumor cell lines, detecting CD107a and cytokine release levels. Loss of CD19 and dilution of CD22 expression is enough to cause blasts to escape from CAR-T surveillance [14, 34]. We then developed an antigen combining strategy in which CD22 CAR-T and CD19 CAR-T cells were sequentially infused. In xenograft models of antigen escape, sequential targeting of CD22 and CD19 therapy demonstrated an improved therapeutic effect when compared with single antigen CAR-T.

Our study further validated the robust clinical antileukemic effects of the novel CD22 CAR-T cells. We combined the CD22 CAR-T incorporating of HIB22 scFv with CD19 CAR-T, which were derived from an HI19a hybridoma and were reported with great clinical efficacy [25], as salvage therapy for the treatment of 4 R/R B-ALL patients. Here, CD22/CD19 sequential therapy was effective in inducing MRD-negative CR in 4/4 (100%) B-ALL patients with moderate CRS, including 3 patients who previously received CD19 CAR-T therapy or blinatumomab and 3 patients with genetic lesions including BCR-ABL, TP53 loss and IKZF1 deletion. Thus, we observed that patients with previous CD19-directed immunotherapy or high-risk genetic lesions could obtain a curative benefit with CD22/CD19 sequential therapy, which is consistent with previously reported clinical experience [41, 42]. Park JH et al. reported that allo-HSCT therapy for patients after CAR-T treatment did not confer a benefit in long-term survival [4], whereas several studies have demonstrated that allo-HSCT after MRD-negative CAR-T therapy significantly mediates durable leukemia-free survival [43, 44]. In our study, CD22/CD19 sequential therapy provided all 4 patients with a time window for allo-HSCT, but limited by economic or other reasons, only patient 1 received allo-HSCT and obtained long-term leukemia-free survival up to 1 year until recent follow-up. Two of three patients who didn’t receive allo-HSCT treatment after CAR-T therapy exhibited relapse-free survival (RFS) for up to 1 year in sequential CD22/CD19 CAR-T trial. In comparison, in our previous clinical study of HI19a derived CD19 CAR-T [25], where the same CD19 CAR-T products were infused as sequential CD22/CD19 CAR-T study, the median RFS in 4 patients who didn’t receive bridged HSCT was 4 months. These data showed that sequential CD22/CD19 CAR-T therapy may effectively prevent leukemia reccurence.

The incidence of CRS following CD19/CD22 dual-targeting CAR-T therapy was variable, with any-grade CRS ranging from 75 to 100% and severe CRS ranging from 6 to 25% [23, 24, 34]. In the present study, we demonstrated the safety profile of CD22/CD19 sequential CAR-T therapy. Three of four patients developed grade 0–1 CRS with mild and self-limiting symptoms. Severe CRS was observed only in patient 1 who developed grade 3 CRS with symptoms of fever, cytopenia and neurotoxic complications. Neurotoxicity has been a common acute toxicity and reported in most CAR-T clinical trials [45–47]. Severe neurotoxicity was observed in up to 13% of patients [3]. In this cohort, no severe neurotoxicity was observed. Only 1/4 patient experienced grade 1 CRES and symptoms were relieved within 2 days. In HI19a derived CD19 CAR-T trial, CRS occurred in 95% (19/20) of patients and the incidence of severe CRS was 45% (9/20) [25]. Compared with single CD19 CAR-T, sequential CD22/CD19 CAR-T therapy demonstrated a safer profile without compromising of efficiency, perhaps due to that the pre-administration CD22 CAR-T had reduced disease burden before infusion of CD19 CAR-T cells. Prior to sequential therapy, patient 1 endured the highest leukemia blasts in bone marrow, which was consistent with previous report that patients with a higher burden of disease might bear more severe CRS. These results addressed the importance of reducing disease burden before CAR-T cells infusion to mitigate toxicity [7, 46, 48].

Analysis of relapsed patient samples revealed that antigen escape and limited persistence of CAR-T cells resulted in a relatively moderate survival benefit. Clinical trials for B-ALL patients have shown that the median disease-free survival achieved in CD19 CAR-T therapy is generally 6–12 months [36, 44]. Despite efficacy of sequential CD19/CD22 CAR-T therapy in inducing CR, 3 patients who didn’t receive bridged HSCT finally relapse. Patient 3 relapsed with CD19^−^CD22^dim^ blasts accompanied by the persistence of a rather low but detectable level of CAR-T cells, thus indicating relapse caused mainly by antigen escape. The other 3 patients achieved CR up to 1 year, but patient 2 and patient 4 finally relapsed with positive or at least partial expression of both CD19 and CD22. This finding is consistent with previous studies showing that CD19 and CD22 doubl-positive relapse occurred in 23/24 patients after dual-targeted immunotherapy [24]. Taken together, efforts should be made not only to combine multiple targets to kill blasts as much as possible, but also to improve the persistence of CAR-T cells to provide sufficient surveillance.

## Conclusions

we constructed a novel CD22 CAR derived from the hybridoma clone HIB22 and validated its antileukemic effects both in vitro and in vivo at the preclinical stage. We further demonstrated the high efficacy and safety profile of sequential therapy combining the novel CD22 CAR-T and CD19 CAR-T in xenograft models and R/R B-ALL patients. Although more investigation is warranted for long-term clinical applicability, these results indicate that CD22/CD19 sequential CAR-T therapy may be an effective and safe approach for preventing antigen escape resistance and improving clinical outcomes in the treatment of B-cell malignancies.

## Supplementary Information

**Additional file 1. **Additional methods.

**Additional file 2: Table S1. **Computational alanine scanning on the complex of extracellular domains 1-3 of hCD22 and HIB22 scFv. **Table S2**. Non-covalent bonds involved in antigen-scFv interaction. **Figure S1.** The expression of CD22 on cell lines and B-ALL patient samples. **Figure S2.** Pathologic analysis,cytokines release in vivo and CAR-T construct and cell lines used in xenograft models. **Figure S3.** Aplasia of B cells, CD19 and CD22 expression in leukemia blasts at pretreatment stage in patients, and T cells subtypes during treatment

## Data Availability

The datasets used during the current study are available from the corresponding author on reasonable request.

## Abbreviations

CAR: Chimeric antigen receptor
R/R B-ALL: Relapsed and/or refractory B cell acute lymphoblastic leukemia
CR: Complete remission
MRD: Minimal residual disease
scFv: Single-chain variable fragment
PDB: Protein data bank
BLAST: Basic local alignment search tool
hCD22: Human CD22
CDR: Complementarity-determining regions
PDF: Pair distribution function
CAMS & PUMC: Chinese Academy of Medical Sciences & Peking Union Medical College
BMMCs: Bone marrow mononuclear cells
E:T: Effector to target
ChiCTR: Chinese clinical trial registration
PBMCs: Peripheral blood mononuclear cells
NCCN: National Comprehensive Cancer Network
T_N_: Naïve T cells
T_CM_: Central memory T cells
T_EM_: Effector memory T cells
T_E_: Effector T cells
RT-qPCR: Real-time quantitative polymerase chain reaction
SFI: Median fluorescence intensity specific to isotype
CRP: C-reactive protein
allo-HSCT: Allogeneic hematopoietic stem cell transplantation
CRS: Cytokine release syndrome
CRES: CAR-T cell related encephalopathy syndrome

## References

[1] Singh AK, McGuirk JP (2020). CAR T cells: continuation in a revolution of immunotherapy. Lancet Oncol.

[2] Lee DW, Kochenderfer JN, Stetler-Stevenson M, Cui YK, Delbrook C, Feldman SA (2015). T cells expressing CD19 chimeric antigen receptors for acute lymphoblastic leukaemia in children and young adults: a phase 1 dose-escalation trial. Lancet.

[3] Maude SL, Laetsch TW, Buechner J, Rives S, Boyer M, Bittencourt H (2018). Tisagenlecleucel in children and young adults with B-cell lymphoblastic leukemia. N Engl J Med.

[4] Park JH, Rivière I, Gonen M, Wang X, Sénéchal B, Curran KJ (2018). Long-term follow-up of CD19 CAR therapy in acute lymphoblastic leukemia. N Engl J Med.

[5] Turtle CJ, Hanafi LA, Berger C, Gooley TA, Cherian S, Hudecek M (2016). CD19 CAR-T cells of defined CD4+:CD8+ composition in adult B cell ALL patients. J Clin Invest.

[6] Gardner R, Finney O, Smithers H, Leger KJ, Annesley CE, Summers C (2016). CD19CAR T cell products of defined CD4:CD8 composition and transgene expression show prolonged persistence and durable MRD-negative remission in pediatric and young adult B-cell ALL. Blood.

[7] Maude SL, Teachey DT, Rheingold SR, Shaw PA, Aplenc R, Barrett DM (2016). Sustained remissions with CD19-specific chimeric antigen receptor (CAR)-modified T cells in children with relapsed/refractory ALL. JCO.

[8] Sotillo E, Barrett DM, Black KL, Bagashev A, Oldridge D, Wu G (2015). Convergence of acquired mutations and alternative splicing of CD19 enables resistance to CART-19 immunotherapy. Cancer Discov.

[9] Gardner R, Wu D, Cherian S, Fang M, Hanafi LA, Finney O (2016). Acquisition of a CD19-negative myeloid phenotype allows immune escape of MLL-rearranged B-ALL from CD19 CAR-T-cell therapy. Blood.

[10] Orlando EJ, Han X, Tribouley C, Wood PA, Leary RJ, Riester M (2018). Genetic mechanisms of target antigen loss in CAR19 therapy of acute lymphoblastic leukemia. Nat Med.

[11] Hegde M, Mukherjee M, Grada Z, Pignata A, Landi D, Navai SA (2016). Tandem CAR T cells targeting HER2 and IL13Rα2 mitigate tumor antigen escape. J Clin Invest.

[12] Grada Z, Hegde M, Byrd T, Shaffer DR, Ghazi A, Brawley VS (2013). TanCAR: a novel bispecific chimeric antigen receptor for cancer immunotherapy. Mol Ther Nucleic Acids..

[13] Tedder TF, Poe JC, Haas KM (2005). CD22: a multifunctional receptor that regulates B lymphocyte survival and signal transduction. Adv Immunol.

[14] Fry TJ, Shah NN, Orentas RJ, Stetler-Stevenson M, Yuan CM, Ramakrishna S (2018). CD22-targeted CAR T cells induce remission in B-ALL that is naive or resistant to CD19-targeted CAR immunotherapy. Nat Med.

[15] Singh N, Frey NV, Engels B, Barrett DM, Shestova O, Ravikumar P (2021). Antigen-independent activation enhances the efficacy of 4–1BB-costimulated CD22 CAR T cells. Nat Med.

[16] Velasco-Hernandez T, Zanetti SR, Roca-Ho H, Gutierrez-Aguera F, Petazzi P, Sánchez-Martínez D (2020). Efficient elimination of primary B-ALL cells in vitro and in vivo using a novel 4–1BB-based CAR targeting a membrane-distal CD22 epitope. J Immunother Cancer.

[17] Nguyen K, Devidas M, Cheng SC, La M, Raetz EA, Carroll WL (2008). Factors influencing survival after relapse from acute lymphoblastic leukemia: a Children's Oncology Group study. Leukemia.

[18] Raetz EA, Cairo MS, Borowitz MJ, Lu X, Devidas M, Reid JM (2015). Re-induction chemoimmunotherapy with epratuzumab in relapsed acute lymphoblastic leukemia (ALL): Phase II results from Children's Oncology Group (COG) study ADVL04P2. Pediatr Blood Cancer.

[19] Chevallier P, Huguet F, Raffoux E, Etienne A, Leguay T, Isnard F (2015). Vincristine, dexamethasone and epratuzumab for older relapsed/refractory CD22+ B-acute lymphoblastic leukemia patients: a phase II study. Haematologica.

[20] Kreitman RJ, Tallman MS, Robak T, Coutre S, Wilson WH, Stetler-Stevenson M (2012). Phase I trial of anti-CD22 recombinant immunotoxin moxetumomab pasudotox (CAT-8015 or HA22) in patients with hairy cell leukemia. J Clin Oncol.

[21] Wayne AS, Kreitman RJ, Findley HW, Lew G, Delbrook C, Steinberg SM (2010). Anti-CD22 immunotoxin RFB4(dsFv)-PE38 (BL22) for CD22-positive hematologic malignancies of childhood: preclinical studies and phase I clinical trial. Clin Cancer Res.

[22] Liu S, Deng B, Lin Y, Yin Z, Pan J, Wu T (2018). Sequential CD19- and CD22-CART cell therapies for relapsed b-cell acute lymphoblastic leukemia after allogeneic hematopoietic stem cell transplantation. Blood.

[23] Spiegel JY, Patel S, Muffly L, Hossain NM, Oak J, Baird JH (2021). CAR T cells with dual targeting of CD19 and CD22 in adult patients with recurrent or refractory B cell malignancies: a phase 1 trial. Nat Med.

[24] Wang N, Hu X, Cao W, Li C, Xiao Y, Cao Y (2020). Efficacy and safety of CAR19/22 T-cell cocktail therapy in patients with refractory/relapsed B-cell malignancies. Blood.

[25] Gu R, Liu F, Zou D, Xu Y, Lu Y, Liu B (2020). Efficacy and safety of CD19 CAR T constructed with a new anti-CD19 chimeric antigen receptor in relapsed or refractory acute lymphoblastic leukemia. J Hematol Oncol.

[26] Grunnet N. Leucocyte typing V; white cell differentiation antigens, vols. 1 and 2: Edited by S.F. Schlossman, L. Boumsell, W. Gilks, J.M. Harlan, T. Kishimoto, C. Morimoto, J. Ritz, S. Shaw, R. Silverstein, T. Springer, T.F. Tedder and R.F. Todd, Oxford University Pr. 1996;384(3):1.

[27] An N, Tao Z, Li S, Xing H, Tang K, Tian Z (2016). Construction of a new anti-CD19 chimeric antigen receptor and the anti-leukemia function study of the transduced T cells. Oncotarget.

[28] Li S, Tao Z, Xu Y, Liu J, An N, Wang Y (2018). CD33-Specific Chimeric Antigen Receptor T Cells with Different Co-Stimulators Showed Potent Anti-Leukemia Efficacy and Different Phenotype. Hum Gene Ther.

[29] Lee DW, Gardner R, Porter DL, Louis CU, Ahmed N, Jensen M (2014). Current concepts in the diagnosis and management of cytokine release syndrome. Blood.

[30] Engel P, Wagner N, Miller AS, Tedder TF (1995). Identification of the ligand-binding domains of CD22, a member of the immunoglobulin superfamily that uniquely binds a sialic acid-dependent ligand. J Exp Med.

[31] Xiao X, Ho M, Zhu Z, Pastan I, Dimitrov DS (2009). Identification and characterization of fully human anti-CD22 monoclonal antibodies. MAbs.

[32] Ereño-Orbea J, Sicard T, Cui H, Mazhab-Jafari MT, Benlekbir S, Guarné A (2017). Molecular basis of human CD22 function and therapeutic targeting. Nat Commun.

[33] Haso W, Lee DW, Shah NN, Stetler-Stevenson M, Yuan CM, Pastan IH (2013). Anti-CD22-chimeric antigen receptors targeting B-cell precursor acute lymphoblastic leukemia. Blood.

[34] Dai H, Wu Z, Jia H, Tong C, Guo Y, Ti D (2020). Bispecific CAR-T cells targeting both CD19 and CD22 for therapy of adults with relapsed or refractory B cell acute lymphoblastic leukemia. J Hematol Oncol.

[35] Maude SL, Frey N, Shaw PA, Aplenc R, Barrett DM, Bunin NJ (2014). Chimeric antigen receptor T cells for sustained remissions in leukemia. N Engl J Med.

[36] Gauthier J, Bezerra ED, Hirayama AV, Fiorenza S, Sheih A, Chou CK (2021). Factors associated with outcomes after a second CD19-targeted CAR T-cell infusion for refractory B-cell malignancies. Blood.

[37] Shah NN, Fry TJ (2019). Mechanisms of resistance to CAR T cell therapy. Nat Rev Clin Oncol.

[38] Xu X, Sun Q, Liang X, Chen Z, Zhang X, Zhou X (2019). Mechanisms of relapse after CD19 CAR T-cell therapy for acute lymphoblastic leukemia and its prevention and treatment strategies. Front Immunol.

[39] Liu S, Deng B, Yin Z, Lin Y, An L, Liu D (2021). Combination of CD19 and CD22 CAR-T cell therapy in relapsed B-cell acute lymphoblastic leukemia after allogeneic transplantation. Am J Hematol.

[40] Tan Y, Cai H, Li C, Deng B, Song W, Ling Z (2021). A novel full-human CD22-CAR T cell therapy with potent activity against CD22(low) B-ALL. Blood Cancer J.

[41] Zhang X, Lu XA, Yang J, Zhang G, Li J, Song L (2020). Efficacy and safety of anti-CD19 CAR T-cell therapy in 110 patients with B-cell acute lymphoblastic leukemia with high-risk features. Blood Adv.

[42] Hu Y, Zhou Y, Zhang M, Ge W, Li Y, Yang L (2021). CRISPR/Cas9-Engineered Universal CD19/CD22 Dual-Targeted CAR-T Cell Therapy for Relapsed/Refractory B-cell Acute Lymphoblastic Leukemia. Clin Cancer Res.

[43] Zhao H, Wei J, Wei G, Luo Y, Shi J, Cui Q (2020). Pre-transplant MRD negativity predicts favorable outcomes of CAR-T therapy followed by haploidentical HSCT for relapsed/refractory acute lymphoblastic leukemia: a multi-center retrospective study. J Hematol Oncol.

[44] Shah NN, Lee DW, Yates B, Yuan CM, Shalabi H, Martin S (2021). Long-Term Follow-Up of CD19-CAR T-Cell Therapy in Children and Young Adults With B-ALL. J Clin Oncol.

[45] Kennedy LB, Salama AKS (2020). A review of cancer immunotherapy toxicity. CA Cancer J Clin.

[46] Schubert ML, Schmitt M, Wang L, Ramos CA, Jordan K, Müller-Tidow C (2021). Side-effect management of chimeric antigen receptor (CAR) T-cell therapy. Ann Oncol.

[47] Morris EC, Neelapu SS, Giavridis T, Sadelain M (2022). Cytokine release syndrome and associated neurotoxicity in cancer immunotherapy. Nat Rev Immunol.

[48] Hirayama AV, Turtle CJ (2019). Toxicities of CD19 CAR-T cell immunotherapy. Am J Hematol.

