# Supplementary material for: A novel and efficient CD22 CAR-T therapy induced a robust antitumor effect in relapsed/refractory leukemia patients when combined with CD19 CAR-T treatment as a sequential therapy
Source: Exp Hematol Oncol. 2022 Mar 22;11:15. doi: 10.1186/s40164-022-00270-5 (PMC8939233; doi:10.1186/s40164-022-00270-5)
Supplement: Supplementary file 1 — Additional file 1. Additional methods. [file 40164_2022_270_MOESM1_ESM.docx]

**Additional file 1 Methods**

**CAR-T cells assessed by flow cytometry**

PBMCs and BMMCs were stained with goat anti-mouse IgG, F(ab’) antibodies incubated at 4°C for 30 minutes. After F(ab’) antibody was washed, the sample was then stained with anti-human CD3 antibody to detect CD22 and CD19 CAR-T cells in total T cells.

**CAR-T cells assessed by quantitative RT-PCR**

Genome DNA was extracted from PBMCs and BMMCs by EasyPure HiPure Plasmid MaxiPrep kit (Transgen, China). Real-Time PCR was performed on 0.2-QuantStudio 5 (Thermo Fisher Scientific, USA). To determine the copy number of CD19 CAR-T and CD22 CAR-T, we designed forward primer, reverse primer and Taqman probe specifically according to different sequence of CD19 scFv and CD22 scFv. To determine copies of CD19 CAR-T, an 8 point standard curve was generated with CD19 CAR plasmids with copy numbers ranging from 5 to 10^6^ added in 50ng non-transduced genomic DNA. Each data point of patient genomic DNA samples and standard samples were evaluated in triplicate and reported as average value. To control the quality of detected DNA, a parallel amplification was performed using 12-20 ng of input genomic DNA and a primer/probe set specific for a non-transcribed genomic sequence upstream of the CDKN1A (p21) gene (GENEBANK: Z85996) (sense primer: 5’- GAAAGCTGACTGCCCCTATTTG-3’, antisense primer: 5’-GAGAGGAAG TGCTGGGAACAAT-3’). Standard curve of p21 gene was produced by dilution of control genomic DNA with 8 point. After amplifications mentioned above, a correction factor (CF) (ng detected/ng input) was produced. The formula to calculate copies of CD19 CAR per microgram genomic DNA was: copies of CD19 CAR/μg DNA =copies calculated from CD19 CAR standard curve/input DNA(ng) ×CF ×1000ng. The accuracy of this assay was determined by the ability to quantify marking of the infused cell product by qPCR.
