# Supplementary material for: A novel and efficient CD22 CAR-T therapy induced a robust antitumor effect in relapsed/refractory leukemia patients when combined with CD19 CAR-T treatment as a sequential therapy
Source: Exp Hematol Oncol. 2022 Mar 22;11:15. doi: 10.1186/s40164-022-00270-5 (PMC8939233; doi:10.1186/s40164-022-00270-5)
Supplement: Supplementary file 2 — Additional file 2: Table S1. Computational alanine scanning on the complex of extracellular domains 1-3 of hCD22 and HIB22 scFv. Table S2. Non-covalent bonds involved in antigen-scFv interaction. Figure S1. The expression of CD22 on cell lines and B-ALL patient samples. Figure S2. Pathologic analysis,cytokines release in vivo and CAR-T construct and cell lines used in xenograft models. Figure S3. Aplasia of B cells, CD19 and CD22 expression in leukemia blasts at pretreatment stage in patients, and T cells subtypes during treatment [file 40164_2022_270_MOESM2_ESM.docx]

**Additional file 2 Tables and Figures**

**Contents**

**Additional file 2: Table 1. Computational alanine scanning on the complex of extracellular domains 1-3 of hCD22 and HIB22 scFv**

**Additional file 2: Table 2. Non-covalent bonds involved in antigen-scFv interaction**

**Additional file 2: Figure 1. The expression of CD22 on cell lines and B-ALL patient samples**

**Additional file 2: Figure 2 Pathologic analysis,cytokines release in vivo and CAR-T construct and cell lines used in xenograft models.**

**Additional file 2: Figure 3. Aplasia of B cells, CD19 and CD22 expression in leukemia blasts at pretreatment stage in patients, and T cells subtypes during treatment.**

**Additional file 2: Table 1. Computational alanine scanning on the complex of extracellular domains 1-3 of hCD22 and HIB22 scFv**

| Mutation | Mutation Energy | Effect of Mutation |
| --- | --- | --- |
| A：TYR 33>ALA | 3.61 | DESTABILIZING |
| A：PHE 199>ALA | 2.3 | DESTABILIZING |
| A：HIS 144>ALA | 2.29 | DESTABILIZING |
| A：LEU 162>ALA | 2.05 | DESTABILIZING |
| A：ARG 139>ALA | 1.61 | DESTABILIZING |
| A：ILE 216>ALA | 1.26 | DESTABILIZING |
| A：LEU 147>ALA | 1.04 | DESTABILIZING |
| A：PRO 149>ALA | 1.03 | DESTABILIZING |
| A：ARG 201>ALA | 0.92 | DESTABILIZING |
| A：PRO 142>ALA | 0.91 | DESTABILIZING |
| A：GLN 146>ALA | 0.9 | DESTABILIZING |
| A：ASP 232>ALA | 0.85 | DESTABILIZING |
| A：PHE 141>ALA | 0.59 | DESTABILIZING |

**Additional file 2: Table 2. Non-covalent bonds involved in antigen-scFv interaction**

| Name | Distance | Category | Type |
| --- | --- | --- | --- |
| R:ARG255:HH12 - A:ASP232:OD2 | 2.4732 | Hydrogen Bond; Electrostatic | Salt Bridge; Attractive Charge |
| R:LYS24:NZ - A:GLU30:OE2 | 3.90625 | Electrostatic | Attractive Charge |
| A:ARG139:NH1 - R:GLU272:OE2 | 4.0759 | Electrostatic | Attractive Charge |
| R:ASP1:HT1 - A:LEU32:O | 2.78374 | Hydrogen Bond | Conventional Hydrogen Bond |
| R:SER28:HN - A:LYS196:O | 2.26942 | Hydrogen Bond | Conventional Hydrogen Bond |
| R:SER28:HG - A:LYS196:O | 2.10981 | Hydrogen Bond | Conventional Hydrogen Bond |
| R:TYR38:HH - A:GLN146:OE1 | 2.53758 | Hydrogen Bond | Conventional Hydrogen Bond |
| R:TRP238:HE1 - A:THR233:O | 2.67814 | Hydrogen Bond | Conventional Hydrogen Bond |
| R:ARG255:HH12 - A:ASP232:OD1 | 2.3693 | Hydrogen Bond | Conventional Hydrogen Bond |
| R:ARG255:HH22 - A:PRO143:O | 2.77205 | Hydrogen Bond | Conventional Hydrogen Bond |
| R:TYR257:HH - A:GLN235:OE1 | 2.06666 | Hydrogen Bond | Conventional Hydrogen Bond |
| R:ASN266:HD22 - A:ASP232:OD1 | 2.21892 | Hydrogen Bond | Conventional Hydrogen Bond |
| A:GLU30:HN - R:SER26:O | 2.1155 | Hydrogen Bond | Conventional Hydrogen Bond |
| A:ARG139:HN - R:GLU272:OE2 | 2.70856 | Hydrogen Bond | Conventional Hydrogen Bond |
| A:ALA164:HN - R:SER109:OG | 2.19473 | Hydrogen Bond | Conventional Hydrogen Bond |
| A:ARG201:HH22 - R:SER32:OG | 1.89184 | Hydrogen Bond | Conventional Hydrogen Bond |
| A:THR233:HG1 - R:ASP264:OD2 | 2.67185 | Hydrogen Bond | Conventional Hydrogen Bond |
| R:ILE2:CA - A:THR31:OG1 | 3.18293 | Hydrogen Bond | Carbon Hydrogen Bond |
| R:LYS24:CE - A:GLU30:OE1 | 3.7597 | Hydrogen Bond | Carbon Hydrogen Bond |
| R:SER235:CB - A:GLN235:OE1 | 3.44772 | Hydrogen Bond | Carbon Hydrogen Bond |
| A:HIS133:CD2 - R:ASP1:O | 3.37505 | Hydrogen Bond | Carbon Hydrogen Bond |
| A:PRO149:CA - R:MET310:O | 3.16814 | Hydrogen Bond | Carbon Hydrogen Bond |
| R:GLU3:OE2 - A:HIS133 | 3.65111 | Electrostatic | Pi-Anion |
| A:LEU162:CD1 - R:TYR31 | 3.3914 | Hydrophobic | Pi-Sigma |
| A:LEU162:CD1 - R:TYR108 | 3.99999 | Hydrophobic | Pi-Sigma |
| R:TYR114 - A:PHE141 | 5.31528 | Hydrophobic | Pi-Pi Stacked |
| R:TYR31 - A:PHE199 | 5.39288 | Hydrophobic | Pi-Pi T-shaped |
| R:MET310 - A:LEU147 | 5.30982 | Hydrophobic | Alkyl |
| R:MET310 - A:ILE151 | 4.77775 | Hydrophobic | Alkyl |
| R:TYR31 - A:ARG201 | 5.29493 | Hydrophobic | Pi-Alkyl |
| R:TRP56 - A:PRO149 | 5.34015 | Hydrophobic | Pi-Alkyl |
| R:TYR108 - A:ALA164 | 4.86628 | Hydrophobic | Pi-Alkyl |
| R:TYR114 - A:PRO142 | 5.04021 | Hydrophobic | Pi-Alkyl |
| R:TYR313 - A:VAL234 | 5.17842 | Hydrophobic | Pi-Alkyl |
| A:TYR33 - R:ILE2 | 5.24851 | Hydrophobic | Pi-Alkyl |
| A:TYR33 - R:PRO115 | 5.30943 | Hydrophobic | Pi-Alkyl |

**Additional file 2: Figure 1**

**
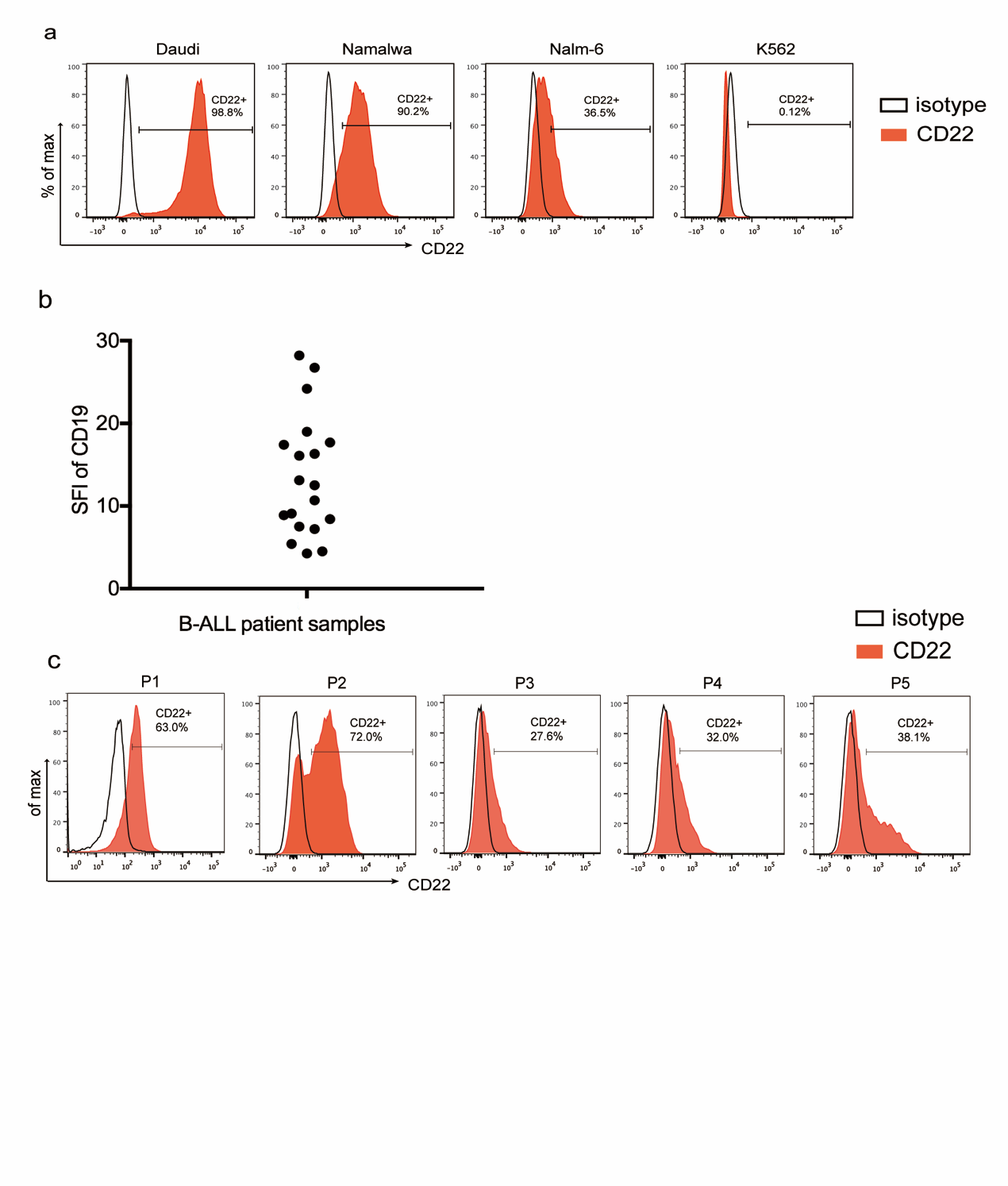
**

**Additional file 2: Figure 1. The expression of CD22 on cell lines and B-ALL patient samples**.

a. CD22 expression on cell lines, including Daudi, Namalwa and Nalm-6 as CD22 positive cell lines and K562 as CD22 negative cells lines.

b. SFI of CD19 on B-ALL patient samples.

c.CD22 expression on BMMCs from 5 B-ALL patients coculture with T cells was assessed by flow cytometry shown in histogram plot.

**Additional file 2: Figure 2. Pathologic analysis,cytokines release in vivo and CAR-T construct and cell lines used in xenograft models.**


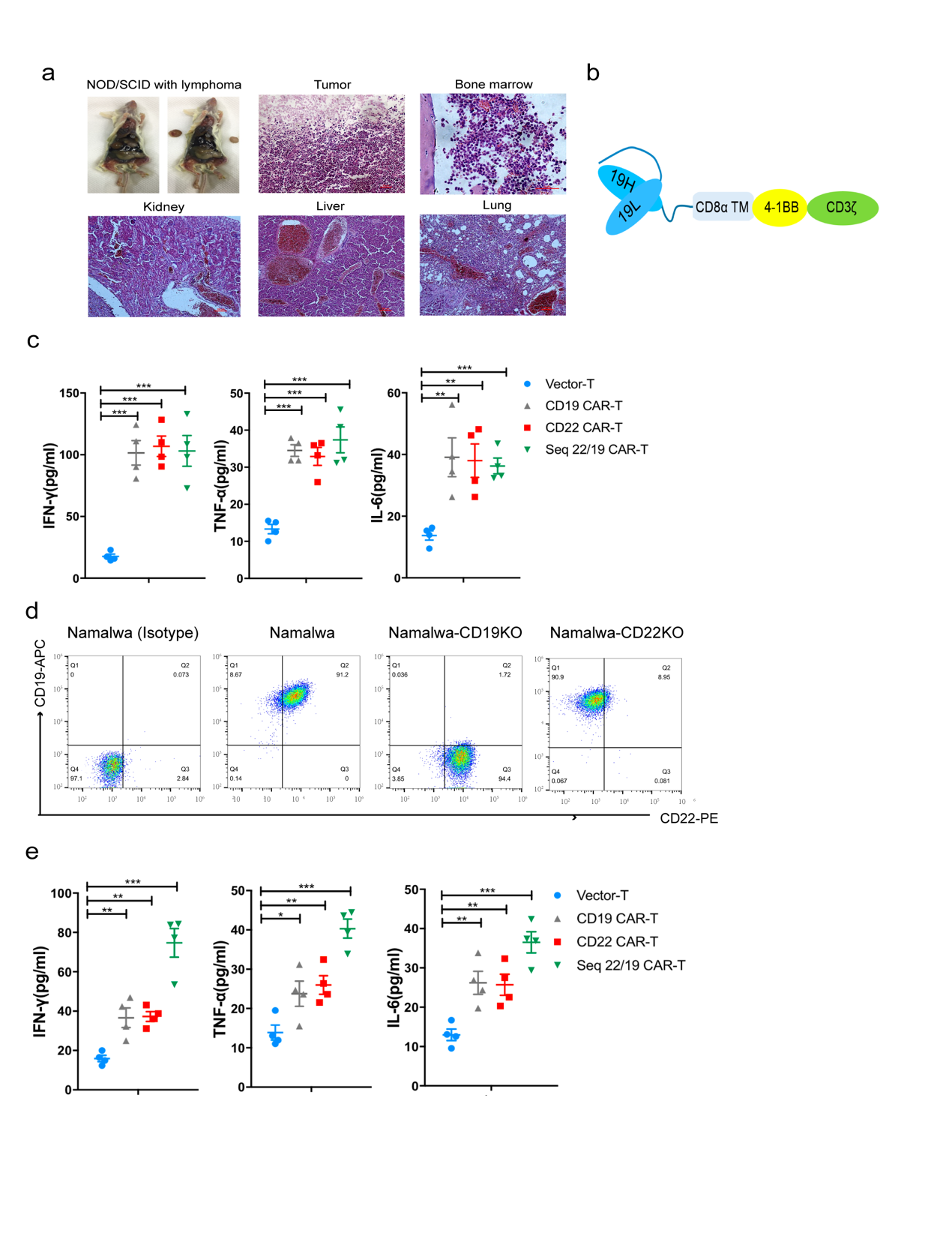


a. Presentative histopathologic analysis of tumor, bone marrow, kidney, liver and lung from Namalwa cells inoculated mice with infusion of vector-T. b.connstruct of HI19a derived CD19 CAR-T. c & e.cytokines release in wild type Namalwa inoculated murine model (c) and antigen relapse murine model (e) on day 6 after tumor cells transpalntation. (n=4 ) d.CD19 and CD22 expression on Namalwa, Namalwa-CD19KO and Namalwa-CD22KO cells assayed by flow cytometry.

**Additional file 2: Figure 3**

**
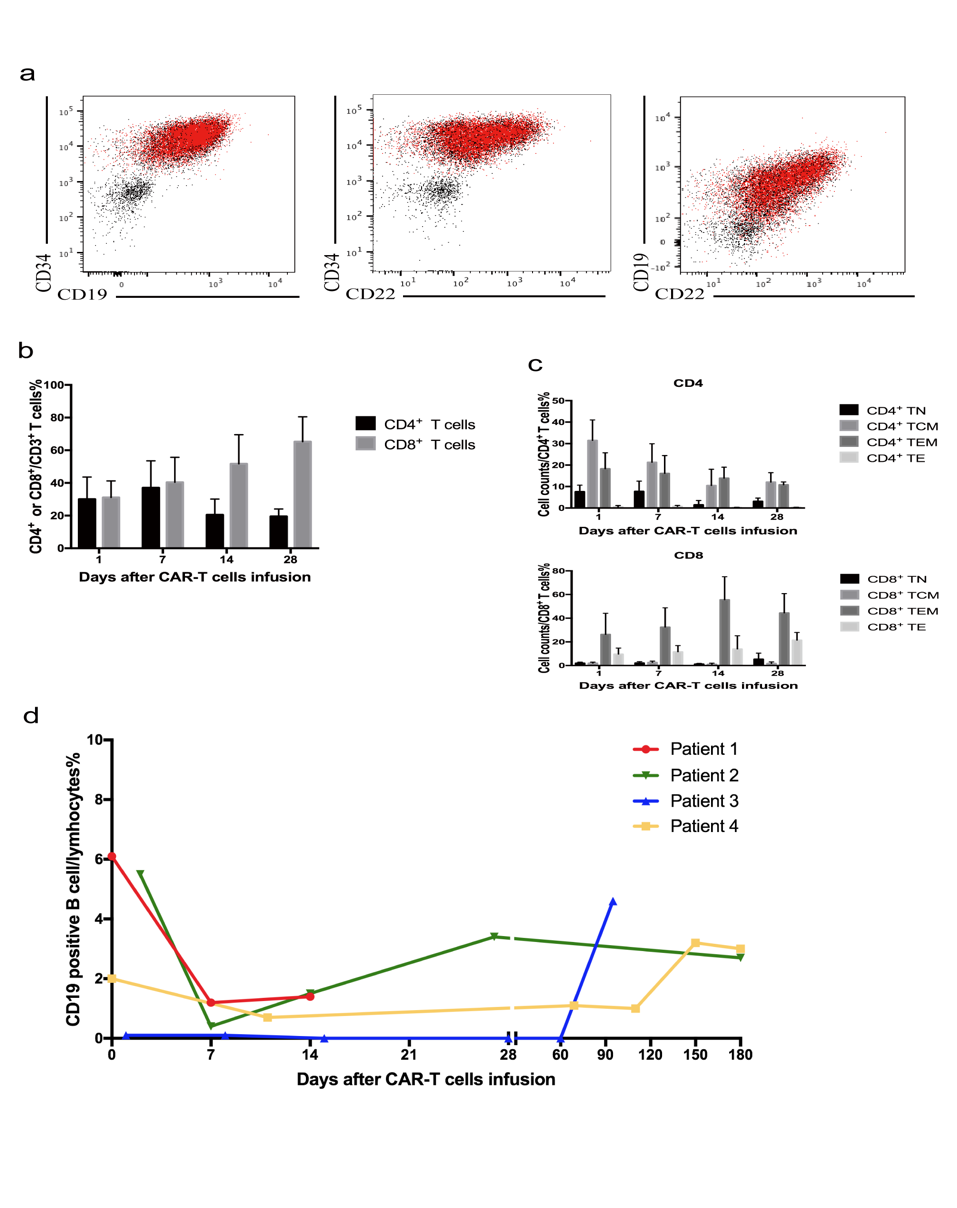
**

**Additional file 2: Figure 3.** **Aplasia of B cells, CD19 and CD22 expression in leukemia blasts at pretreatment stage in patients, and T cells subtypes during treatment.**

a. CD19 and CD22 expression on leukemia blasts in patient 1 before CAR-T cells infusion.

b. Ratio of CD4 or CD8 positive T cells in total CD3 positive T cells was detected, which revealed a decrease in percentage of CD4 positive subtype accompanied by an increase in CD8 positive population.

c. Circulating T cells in peripheral blood were subtyped into naïve T cells (T_N_, CD45RA^+^ CCR7^+^), central memory T cells (T_CM_, CD45RA^−^ CCR7^+^), effector memory T cells (T_EM_, CD45RA^−^ CCR7^−^), and effector T cells (T_E_, CD45RA^+^ CCR7^−^) via flow cytometry. CD8^+^ T_EM_ and T_E_ significantly expanded after CAR-T cells infusion.

d. Percentage of CD19 positive B cells in total lymphocytes in peripheral blood during sequential CAR-T treatment.
